# Development of the Japanese Version of the State Self-Compassion Scale (SSCS-J)

**DOI:** 10.3389/fpsyg.2021.779318

**Published:** 2022-01-14

**Authors:** Yuki Miyagawa, István Tóth-Király, Marissa C. Knox, Junichi Taniguchi, Yu Niiya

**Affiliations:** ^1^Faculty of Psychology, Otemon Gakuin University, Osaka, Japan; ^2^Department of Psychology, Concordia University, Montréal, QC, Canada; ^3^Department of Educational Psychology, The University of Texas at Austin, Austin, TX, United States; ^4^Department of Psychology, Tezukayama University, Nara, Japan; ^5^Department of Global and Interdisciplinary Studies, Hosei University, Tokyo, Japan

**Keywords:** self-compassion, bifactor model, exploratory structural equation modeling, self-compassionate mindstate induction, construct validity

## Abstract

Research in the U.S. developed and validated the State Self-Compassion Scale (SSCS), which measures self-compassionate reactions toward a specific negative event. The current study is aimed at developing the Japanese version of the State Self-Compassion Scale (SSCS-J) and extending previous findings in the U.S. by showing measurement invariance across sexes and demonstrating the construct validity of this scale. Across two studies (*n* = 596 in Study 1, *n* = 474 in Study 2), the bifactor exploratory structural equation modeling representation of the SSCS-J showed excellent fit in which a single global factor (i.e., self-compassion) and most of the specific factors (six subscales) were well defined. Study 1 further provided evidence for the measurement invariance across sexes. The SSCS-J was related with higher trait self-compassion and lower fear of and negative beliefs about self-compassion. In Study 2, participants who were instructed to be self-compassionate reported higher scores in the SSCS-J relative to those in the control condition. These results attest to the replicability of the factor structure of the SSCS in Japan and provide further evidence for the construct validity of this scale.

## Introduction

Cumulative evidence shows that self-compassion (i.e., treating oneself with kindness) has psychological benefits when people suffer from personal struggles or life situations (Barnard and Curry, 2011; Breines and Chen, 2012; Neff et al., 2018; Ewert et al., 2021). Neff (2003) and Neff et al. (2018, 2019) proposed that self-compassion comprises a dynamic psychological system determined by compassionate self-responding and reduced uncompassionate self-responding. Specifically, self-compassion includes (a) self-kindness rather than self-judgment, (b) common humanity rather than isolation, and (c) mindfulness rather than over-identification (Neff, 2003; Neff et al., 2018, 2019). Self-kindness involves being understanding of and genuinely caring for the self. Common humanity helps people see the connection between their own and others’ experiences and recognize that no one is perfect. Mindfulness entails paying balanced attention to and seeing the big picture of their experiences. These three components are subsumed under compassionate self-responding (Neff et al., 2018, 2019). People high in trait self-compassion (hereinafter, self-compassionate people) also show reduced uncompassionate self-responding: they avoid being harshly judgmental of themselves, feeling alone in their suffering, and overreacting to their experiences (Neff, 2003; Neff et al., 2018, 2019). Taken together, the level of self-compassion is determined by the psychological balance between compassionate self-responding (self-kindness, common humanity, and mindfulness) and reduced uncompassionate self-responding (reduced self-judgment, isolation, and over-identification; Neff, 2003; Neff et al., 2018, 2019).

The majority of research has focused on trait levels of self-compassion and how people care for the self in times of suffering *in general* (Neff, 2003; Barnard and Curry, 2011; Neff et al., 2018). The literature reveals that trait self-compassion is strongly and consistently related to positive psychological functioning such as having high levels of mental health (MacBeth and Gumley, 2012) and well-being (Zessin et al., 2015), using adaptive emotion regulation (Sirois et al., 2019) and coping strategies (Ewert et al., 2021), and creating good relationships with others (Lathren et al., 2021). In contrast, very little research has examined whether people are actively self-compassionate at the moment they experience a specific negative event (i.e., state self-compassion; Neff et al., 2021). It is likely that self-compassionate people might not always engage in self-compassion when confronted with a stressful situation for a number of reasons. For example, even people who are self-compassionate *in general* might not be able to keep treating themselves compassionately when they encounter multiple or sequential stressors that deplete the limited psychological resources for self-regulation (Muraven and Baumeister, 2000). Hence, it is important to examine the similarities and differences of trait and state self-compassion for advancing the understanding of how self-compassion functions and relates to positive psychological functioning.

Neff et al. (2021) introduced and validated a new scale to measure state self-compassion (the State Self-Compassion Scale, SSCS). They rewrote and created 18 items from the Self-Compassion Scale (SCS) in the present tense so that each item represents the current self-compassionate response toward a specific event (see section “Measures” and Supplementary Table 1, for more). Across three studies, using the bifactor exploratory structural equation modeling (bifactor ESEM) framework (Morin et al., 2016, 2020), Neff et al. (2021) confirmed that, consistent with the SCS, the bifactor ESEM representation (incorporating one global factor representing self-compassion) of state self-compassion was superior to other representations such as two bifactor-ESEM (incorporating two global factors representing compassionate and uncompassionate self-responding; Tóth-Király et al., 2017; Neff et al., 2019). Neff et al. (2021) also validated the short form of the SSCS, which consisted of six items adapted from the 18 items version of the SSCS. Furthermore, Neff et al. (2021, Studies 2 and 3) showed that people who are induced to be self-compassionate are higher in the SSCS and its short form than those in the control group. These results attest to the validity of the SSCS and this scale is considered the first validated measure of state self-compassion.

However, several issues remain unsolved. First, it is unclear if this scale can be used in a different cultural context, such as in Japan. Given that self-compassion is a positive psychological resource across cultures (e.g., Miyagawa et al., 2015; Neff et al., 2018), the translation of the SSCS would contribute to understanding whether people in different cultures would take similar self-compassionate responses toward situations. Second, the construct validity of the SSCS should be examined in more detail, including the associations between the SCS and the SSCS.

To fill in these research gaps, we developed the Japanese version of the State Self-Compassion Scale (SSCS-J) and examined its construct validity. In two studies, we tested whether the bifactor ESEM approach to the SSCS (Neff et al., 2021) would also be supported in Japan. This analytic framework makes it possible to take into account two sources of construct-relevant psychometric multidimensionality that are typically present in multidimensional measures. First, this framework accounts for the co-existence of hierarchically ordered constructs by providing a direct and explicit estimate of global self-compassion (i.e., G-factors; Reise, 2012) in conjunction with specific factors (i.e., S-factors) depicting the unique quality associated with each subscale and left unexplained by the global factors. Second, this framework also accounts for the fallible nature of indicators used to assess each construct by allowing cross-loadings to be freely estimated among all factors used to reflect self-compassion (Morin et al., 2020).

**FIGURE 1:**
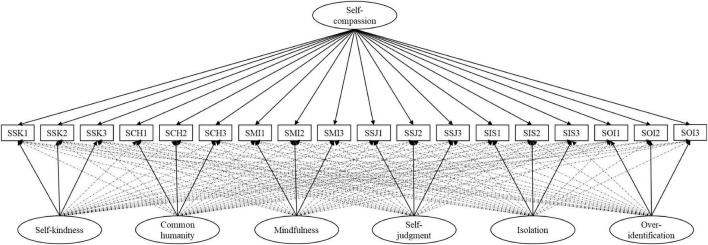
Graphical depiction of the bifactor ESEM representation of self-compassion. SSK, state self-kindness; SSJ, state self-judgment; SCH, state common humanity; SIS, state isolation; SMI, state mindfulness; SOI, state over-identification. Ovals represent latent variables and squares represent questionnaire items. Unidirectional solid lines represent target paths, unidimensional dashed lines represent cross-loadings.

Following Neff et al. (2021), we assessed the model fit of the proposed representation of the SSCS (Figure 1) relative to the eight alternative models (see Supplementary Figure 1). We expected that the factor structure of the SSCS would be replicated in Japan because the SCS, from which the state measure is created, is equivalent across cultures (Tóth-Király and Neff, 2021). Additionally, on the basis of Neff et al. (2021), we examined whether the experimental manipulation of self-compassion increased state self-compassion.

Furthermore, we extended Neff et al.’s (2021) findings in three ways. First, we examined the measurement invariance of the bifactor-ESEM representation of SSCS across sex groups in Study 1. Since Tóth-Király and Neff (2021) reported that the SCS was equivalent for men and women, we expected that the SSCS-J would also be equivalent across these groups.

Second, to show the construct validity of the SSCS-J, we examined associations between the SSCS-J and other relevant variables in Study 1. Given that both the SSCS-J and the SCS-J measure different aspects (i.e., state versus trait) of the same concept, we expected these two scales to be strongly correlated. State self-compassion was also expected to be associated with a lower level of threat appraisal and a higher level of controllability appraisal of the situation given that people who display state self-compassion may take a broader and more balanced perspective of the situation without amplifying or diminishing their experience (Neff, 2003; Neff et al., 2018, 2021). Similar to the previous findings (Zessin et al., 2015; Neff et al., 2018, 2021; Sirois et al., 2019), state self-compassion was hypothesized to correlate positively with positive affect and negatively with negative affect. We further focused on the associations between state self-compassion and fear of self-compassion as well as negative beliefs about self-compassion. People who fear that becoming self-compassionate shows personal weaknesses and makes them feel sad and empty (i.e., fear of self-compassion; Gilbert et al., 2011) may be less likely to use self-compassion during negative events. Similarly, holding misbeliefs that self-compassion leads to complacency, self-indulgence, and a lack of self-responsibility (i.e., negative beliefs about self-compassion; Chwyl et al., 2020) may deter people from using self-compassion. Therefore, state self-compassion was expected to be inversely related to fear of self-compassion and negative beliefs about self-compassion.

Third, previous work (Neff et al., 2021) has not considered the differences between trait and state self-compassion in relation to other variables. We expected state self-compassion to show stronger associations with the situational variables (i.e., cognitive appraisals and affect after recalling a situation) whereas trait self-compassion would exhibit stronger relationships with dispositional fear of and negative beliefs about self-compassion. We also explored whether the experimental manipulation of self-compassion in a difficult situation would be only effective for boosting state self-compassion or whether it has an impact on trait self-compassion as well. These examinations would clarify the unique characteristics of state self-compassion relative to trait self-compassion.

## Study 1

In Study 1, we aimed to test the factor structure of the SSCS-J and its measurement invariance across sex groups. We also attempted to provide the validity of this scale by showing its associations with trait self-compassion, cognitive appraisals, affect, and fear of and negative beliefs about self-compassion.

### Method

#### Participants

Following the sample size reported in Neff et al. (2021, Study 1, *n* = 588) as well as *a priori* guidelines about sample sizes necessary for factor analyses (e.g., de Winter et al., 2009; Comrey and Lee, 2013), we recruited 600 participants in their twenties to fifties from a Japanese Internet research company, Rakuten Insight, which has about 2.2 million registered monitors across Japan. Data collection continued until a balanced sex proportion was reached. We excluded the data of 4 participants from the initial sample because they reported no current personal experience of suffering. Thus, the final sample comprised 596 participants (296 men, 300 women; *M*_age_ = 40.0, *SD* = 11.1).

#### Procedure

First, participants read an informed consent form in the web survey, and those who agreed to participate clicked the box embedded in the consent form. Subsequently, participants were asked to recall and briefly describe a personal experience in which they currently felt pain or suffering, and then they completed the SSCS-J and the measures of cognitive appraisal of the situation and their current mood. Afterward, participants answered the trait measures. At the end of this study, participants were debriefed and thanked for their participation. This research was approved by the Institutional Review Board of the affiliated university of the first author.

#### Measures

##### Japanese Version of the State Self-Compassion Scale

With the permission of the original author, we developed the Japanese version of the SSCS. Whereas the SSCS has been created based on the SCS, several new items were added. To ensure equivalence across languages, we translated the SSCS using the back-translation procedure. Specifically, the first author translated this scale into Japanese in consultation with the fourth author. The fifth author, who is fluent in English and specializes in social and cultural psychology, back-translated it into English without seeing the original items. Then, the third author, who is also an original author of Neff et al. (2021), checked the equivalence of the original and back-translated items. When there were some inconsistencies between the original and back-translated items, The first, third, and fifth authors discussed and resolved them. The second author, who is fluent in English and has expertise in scale development, also checked the original and back-translated items. All the authors confirmed the final version of the SSCS-J.

The SSCS-J has 18 items to measure state self-compassion. Each item represents participants’ current level of self-compassionate in a painful situation. Example items are “I’m giving myself the caring and tenderness I need,” and “I see my difficulties as part of life that everyone goes through.” Among them, nine items from the self-judgment, isolation, and over-identification subscales are reverse-coded to indicate the relative absence of these types of uncompassionate self-responding.

Participants were instructed to indicate the degree to which each item described their responses toward their recalled situation on a 5-point scale ranging from 1 (*not at all true for me*) to 5 (*very true for me*). Supplementary Table 1 shows the instruction and items of the Japanese translation of this scale.

##### Cognitive Appraisals of the Recalled Situation

Participants completed the measure of the current cognitive appraisals of their recalled situation (Kato, 2001) on a 5-point scale ranging from 1 (*not at all true for me*) to 5 (*very true for me*). This scale consists of three subscales: controllability (four items such as “I can change the situation”), threat (three items such as “I think that this situation is painful”), and importance appraisals (two items such as “I think that this situation has an important influence on me”). Following Kato (2001), we averaged items of each subscale to create controllability appraisal (α = 0.878), threat appraisal (α = 0.859), and importance appraisal (α = 0.791).

##### Positive and Negative Affect

Participants reported their current feelings by responding to the Japanese version of the PANAS (Kawahito et al., 2011) on a 6-point scale ranging from 1 (*not at all true for me*) to 6 (*very true for me*). We averaged 10 positive adjectives (e.g., “active”) as positive affect (α = 0.858) and 10 negative adjectives (e.g., “afraid”) as negative affect (α = 0.821).

##### Fear of Self-Compassion

To assess the trait levels of participants’ fear of being self-compassionate, we used the Japanese version of Fear of Compassion for Self Scale (Asano et al., 2017). Note that whereas the original version (Gilbert et al., 2011) has one factor, the Japanese version comprises two subscales: miserable with self-compassion and demerits of self-compassion. Examples of each subscale are “When I try and feel kind and warm to myself, I just feel kind of empty,” (miserable with self-compassion) and “I fear that if I develop compassion for myself, I will become someone I do not want to be” (demerits of self-compassion). Participants were asked to indicate the degree to which each item described their general tendency to feel fear of self-compassion on a 5-point scale ranging from 1 (*not at all true for me*) to 5 (*very true for me*). Following the previous study (Asano et al., 2017), we averaged items of each subscale to represent miserable with self-compassion (α = 0.886) and demerits of self-compassion (α = 0.918).

##### Negative Beliefs About Self-Compassion

We translated the scale to measure negative beliefs about self-compassion (Chwyl et al., 2020) using the back-translation procedure. This scale has 10 items that represent misbeliefs that self-compassion leads to complacency, self-indulgence, and a lack of self-responsibility (Chwyl et al., 2020). Example items are “When I’m kind to myself, I’ll behave more self-indulgently,” and “When I’m understanding of my mistakes, I’m less likely to fix them.” Participants were asked to indicate the degree to which each item described their general beliefs about self-compassion on a 5-point scale ranging from 1 (*not at all true for me*) to 5 (*very true for me*). Following the previous study (Chwyl et al., 2020), the 10 items were averaged to create negative beliefs about self-compassion (α = 0.938).

##### Trait Self-Compassion

We used the Japanese version of the Self-Compassion Scale (the SCS-J; Miyagawa et al., 2015) to assess the trait levels of self-compassion on a 5-point scale ranging from 1 (*not at all true for me*) to 5 (*very true for me*). This scale has six subscales that represent the presence of compassionate self-responding (i.e., self-kindness, common humanity, and mindfulness) and the absence of uncompassionate self-responding (i.e., the reduced self-judgment, isolation, and over-identification). Unlike the SSCS, this scale assesses the general tendency to engage in self-compassion when personal suffering occurs (e.g., “I try to be loving toward myself when I’m feeling emotional pain”). Similar to the SSCS, we reverse-coded the items of the self-judgment, isolation, and over-identification subscales. Thus, higher scores of these subscales represent the absence of uncompassionate self-responding. We computed mean scores of each subscale as self-kindness (α = 0.815), self-judgment (α = 0.747), common humanity (α = 0.734), isolation (α = 0.819), mindfulness (α = 0.709), and over-identification (α = 0.778). We also created a self-compassion total score by averaging all 26 items after reverse-coding the items of the self-judgment, isolation, and over-identification subscales (α = 0.879).

### Data Analyses

#### Measurement Models

To test the factor structure of the SSCS-J, we contrasted and compared nine alternative models following Neff et al. (2021): one factor CFA, two-factor CFA and ESEM (representing two correlated global factors of compassionate and reduced uncompassionate self-responding); six-factor CFA and ESEM (representing the six components of self-compassion); bifactor CFA and ESEM (representing a global self-compassion factor and its six components); and two bifactor CFA and ESEM (representing two correlated global factors of compassionate and reduced uncompassionate self-responding and the six specific components). All models were estimated using M*plus* 7.4 (Muthén and Muthén, 2015) with the weighted least squares mean- and variance-adjusted estimator (WLSMV) which has been found to outperform maximum-likelihood estimation methods for ordered-categorical items following asymmetric response thresholds (Finney and DiStefano, 2013). In CFA, scale items only loaded on their target factors, cross-loadings were constrained to zero but correlations between the factors were freely estimated. ESEM models were estimated the same way as CFA models but cross-loadings between items and non-target factors were freely estimated and targeted to be as close to zero as possible *via* an oblique target rotation procedure (Browne, 2001). In bifactor-CFA, all items were allowed to simultaneously load on one or two general factors (G-factor) and on one *a priori* specific factor (S-factor), no cross-loadings were integrated, while the factors were orthogonal to one another and not allowed to correlate. The bifactor-ESEM solutions were estimated the same way as the bifactor-CFA models, but cross-loadings were freely estimated among the S-factors and “targeted” to be close to zero *via* an orthogonal target rotation. In bifactor models including two global factors, these factors were allowed to correlate.

#### Tests of Measurement Invariance

We also tested the measurement invariance of the SSCS-J across sexes. This was achieved by gradually imposing equality constraints on the various model parameters in the following order (Millsap, 2011): (a) configural invariance (equal factor structure), (b) weak invariance (equal factor loadings), (c) strong invariance (equal item thresholds), (d) strict invariance (equal item uniquenesses), (e) invariance of variance–covariance matrix (equal factor variances and covariances), and (f) invariance of latent means (equal latent factor means).

#### Model Evaluation

The adequacy of all models was assessed using sample-size-independent fit indices: the comparative fit index (CFI), the Tucker–Lewis index (TLI), and the root mean square error of approximation (RMSEA). Based on commonly used guidelines (Hu and Bentler, 1999; Yu, 2002; Marsh et al., 2005), values greater than 0.90 and 0.95 for the CFI and TLI were, respectively, taken to reflect adequate and excellent fit, whereas values smaller than 0.08 or 0.06 for the RMSEA were, respectively, taken to indicate acceptable and excellent fit. To compare the nested measurement invariance models, we examined the relative changes (Δ) in CFI, TLI, and RMSEA: a decrease in CFI and TLI of 0.010 or higher or an increase in RMSEA of 0.015 or higher indicate a lack of invariance or a lack of similarity (Cheung and Rensvold, 2002; Chen, 2007).

As model evaluation should not be based solely on fit indices (Marsh et al., 2004; Morin et al., 2016), these alternative models were also compared following recommendations formulated by Morin et al. (2016, 2020). We first contrasted the first-order CFA and ESEM models where ESEM should be retained in case of: (1) improved model fit, (2) equally well-defined factors with similarly strong main loadings, (3) cross-loadings with reasonable magnitude, and (4) decreased estimates of factor correlations in ESEM relative to CFA (Morin et al., 2016, 2020). The retained first-order solution was then contrasted with its bifactor counterpart. In this comparison, support for the bifactor solution would come from the observation of: (1) similar level of fit, (2) a well-defined G-factor, and (3) at least a subset of well-defined S-factors (Morin et al., 2016, 2020).

#### Reliability

To assess reliability, we reported Cronbach’s alpha coefficient (α) and McDonald’s model-based composite reliability (CR) index. Following Perreira et al. (2018), we considered CRs above 0.50 to be satisfactory. Given that we tested bifactor models, we also calculated both omega (ω) and omega hierarchical (ω_H_) indices (Brunner et al., 2012; Rodriguez et al., 2016). Omega represents the percentage of variances in the self-compassion total score explained by both G-factor and six S-factors, while omega hierarchical describes the percentage of variances in this total score accounted for only by the G-factor. To determine the amount of reliable variance in the total score that is not explained by error, we divided ω_H_ by ω and the use of the total score is justified when this value is above 0.75 (see Reise et al., 2013). To estimate the remaining reliable variance attributed to S-factors, ω_H_ is subtracted from ω (Rodriguez et al., 2016).

#### Further Tests of Construct Validity

To examine the construct validity of the SSCS-J, we computed Pearson’s product-moment correlations between the SSCS-J and the other relevant variables, using IBM SPSS Statistics (Version 27). Effect sizes were evaluated according to thresholds established by Cohen (1988): correlations were considered small between 0.10 and 0.30, medium between 0.31 and 0.50, and large over 0.51. Furthermore, we used M*plus* 7.4 (Muthén and Muthén, 2015) and conducted a path analysis to clarify the unique relations of state and trait self-compassion to cognitive appraisals, affect, and fear of and negative beliefs about self-compassion. In the path model, we entered state and trait self-compassion as predictors and cognitive appraisals, affect, and fear of negative beliefs about self-compassion as outcome variables. This model allowed us to examine whether state self-compassion would be related to other variables beyond trait self-compassion.

### Results and Brief Discussion

#### Alternative Representations of the Japanese Version of the State Self-Compassion Scale

Table 1 shows the goodness-of-fit indices for the nine alternative models. One-factor and two-factor models failed to achieve a good fit. The fit of the six-factor CFA solution was good, although the six-factor ESEM model had a substantially improved fit (ΔCFI = +0.038, ΔTLI = +0.030, ΔRMSEA = −0.020). Regarding the bifactor solutions of the SSCS-J, the bifactor ESEM model had better fit than the bifactor (ΔCFI = +0.101, ΔTLI = +0.114, ΔRMSEA = −0.061) and two-bifactor (ΔCFI = +0.041, ΔTLI = +0.036, ΔRMSEA = −0.025) CFA solutions, while the difference in fit of the bifactor and two-bifactor ESEM models was negligible (ΔCFI = +0.003, ΔTLI = +0.009, ΔRMSEA = −0.008).

**TABLE 1:** Goodness-of-fit indices for the estimated solution for the Japanese version of the State Self-Compassion Scale in Study 1.

Models	χ^2^	df	CFI	TLI	RMSEA [90% CI]
One factor CFA	2785.098*	135	0.667	0.622	0.181 [0.176, 0.187]
Two-factor CFA	1649.180*	134	0.809	0.782	0.138 [0.132, 0.144]
Two-factor ESEM	1484.111*	118	0.828	0.777	0.139 [0.133, 0.146]
Six-factor CFA	533.907*	120	0.948	0.934	0.076 [0.070, 0.083]
Six-factor ESEM	172.780*	60	0.986	0.964	0.056 [0.046, 0.066]
Bifactor CFA	994.282*	117	0.890	0.856	0.112 [0.106, 0.119]
Bifactor ESEM	123.124*	48	0.991	0.970	0.051 [0.040, 0.063]
Two-bifactor CFA^a^	514.842*	116	0.950	0.934	0.076 [0.069, 0.083]
Two-bifactor ESEM	85.552*	41	0.994	0.979	0.043 [0.030, 0.055]

*p < 0.05.

*CFA, confirmatory factor analysis; ESEM, exploratory structural equation modeling; CFI, comparative fit index; TLI, Tucker–Lewis index; RMSEA, root-mean square error of approximation; CI, confidence interval.*

*^a^The residual covariance matrix was not positive definite.*

Since the six-factor CFA and ESEM models achieved a good fit, we first examined these models in more details. Examination of parameter estimates (reported in Supplementary Table 2) revealed that the factors were well-defined in both the six-factor CFA (λ = 0.560–0.842, *M* = 0.740) and ESEM (λ = 0.316–0.959, *M* = 0.609) solutions. Although multiple statistically significant cross-loadings were present in the ESEM model, only items SJ2 (“*I’m being a bit cold-hearted towards myself*”) and OI1 (“*I’m obsessing and fixating on everything that’s wrong*”) had a cross-loading that was higher than their target loadings. Overall, the presence of the cross-loadings did not undermine the definition of the factors, yet they reinforce the need to explicitly take into account this source of construct-relevant psychometric multidimensionality. Finally, factor correlations were substantially reduced in the ESEM (*r* = 0.067–0.619, *M* = 0.318) relative to the CFA (*r* = 0.100–0.902, *M* = 0.493) solution. Therefore, the six-factor ESEM model was retained.

Next, we incorporated one (representing self-compassion) or two (representing compassionate and uncompassionate self-responding) G-factors into the six-factor ESEM model. Examining the parameter estimates in the two-bifactor ESEM model (Supplementary Table 3) shows that the two global factors were weakly defined by the majority of their loadings (compassionate self-responding: λ = −0.278–0.616, *M* = 0.237; uncompassionate self-responding: λ = −0.507–0.188, *M* = 0.165), arguing against the need to incorporate a second G-factor.

We thus retained the bifactor-ESEM model in Figure 1 as the optimal representation of state self-compassion, a conclusion that was also supported by the examination of parameter estimates (reported in Table 2) which revealed a well-defined self-compassion G-factor (λ = 0.125–0.777, *M* = 0.519). Beyond this G-factor, the self-kindness (λ = 0.537–0.657, *M* = 0.607), common humanity (λ = 0.562–0.832, *M* = 0.710), mindfulness (λ = 0.353–0.578, *M* = 0.502) and isolation (λ = 0.240–0.639, *M* = 0.465) S-factors retained a higher amount of specificity, whereas the self-judgment (λ = 0.136–0.285, *M* = 0.215) and over-identification (λ = −0.064–0.373, *M* = 0.205) factors retained a lower amount of specificity in the presence of the G-factor.

**TABLE 2:** Standardized factor loadings for the bifactor-ESEM solution for the Japanese version of the State Self-Compassion Scale in Study 1.

	SC (λ)	SK (λ)	SJ (λ)	CH (λ)	IS (λ)	MI (λ)	OI (λ)
SSK1	0.440**	0.537**	–0.113	0.027	−0.165**	0.011	−0.204*
SSK2	0.493**	0.657**	−0.127*	0.048	–0.074	0.110**	–0.092
SSK3	0.371**	0.628**	0.298**	0.174**	0.049	0.271**	0.091
SSJ1	0.777**	–0.025	**0.285**	–0.052	–0.008	−0.076*	−0.173*
SSJ2	0.679**	0.253**	**0.136**	–0.035	0.082*	−0.260**	0.035
SSJ3	0.685**	0.067	0.225**	−0.128**	0.067	–0.021	0.146*
SCH1	0.293**	0.090*	−0.108*	0.562**	0.119**	0.056	–0.040
SCH2	0.125**	0.039	0.082**	0.832**	−0.090**	0.062*	–0.023
SCH3	0.170**	0.097**	−0.076*	0.736**	0.033	0.127**	–0.003
SIS1	0.675**	−0.102**	0.024	0.002	0.639**	0.006	−0.082**
SIS2	0.604**	−0.155**	–0.046	0.059*	0.240**	−0.089*	0.179**
SIS3	0.634**	–0.007	0.039	0.001	0.517**	0.059*	0.044
SMI1	0.558**	0.275**	–0.066	0.113**	0.019	0.353**	0.059
SMI2	0.495**	0.149**	−0.120**	0.154**	–0.005	0.576**	–0.025
SMI3	0.405**	0.114**	0.052	0.083**	0.016	0.578**	0.058
SOI1	0.722**	−0.136**	0.140	−0.078**	−0.096*	0.005	−**0.064**
SOI2	0.530**	−0.156**	0.019	−0.102**	0.036	−0.100**	0.179*
SOI3	0.686**	−0.252**	–0.017	−0.069*	0.002	0.156**	0.373**

**p < 0.01, *p < 0.05.

*λ, standardized factor loading; ESEM, exploratory structural equation modeling; SC, self-compassion; SSK, state self-kindness; SSJ, state self-judgment; SCH, state common humanity; SIS, state isolation; SMI, state mindfulness; SOI, state over-identification.*

*Target loadings are bolded. Items of the self-judgment, isolation, and over-identification subscales were reverse-coded.*

Reliability indicators (reported in Table 3) show that Cronbach’s alpha and CR were excellent for the total score. When looking at the subscales, most had adequate levels of reliability (αs ≥ 0.725, CRs ≥ 0.635) except for self-judgment and over-identification which demonstrated low levels of model-based CR. The total score in the bifactor ESEM model had sufficient omega (ω = 0.935) and omega hierarchical (ω_H_ = 0.815) coefficients, indicating that 87.1% (ω_H/_ω) of the variances in the total score is explained by the self-compassion G-factor, while 12.0% could be attributed to the S-factors over and above the G-factor.

**TABLE 3:** Reliabilities of the SSCS-J and reliable variance of the total score explained by the G-factor and S-factors in the bifactor ESEM solution for the SCS-J.

	α	CR	ω	ω_H_	GF	SF
**Study 1**						
State self-compassion	0.866	0.926	0.935	0.815	0.871	0.120
State self-kindness	0.747	0.772	0.837	–	–	–
State common humanity	0.737	0.787	0.799	–	–	–
State mindfulness	0.725	0.635	0.771	–	–	–
State self-judgment	0.752	0.277	0.821	–	–	–
State isolation	0.785	0.667	0.852	–	–	–
State over-identification	0.670	0.218	0.753	–	–	–
**Study 2 pretest**						
State self-compassion	0.897	0.950	0.976	0.905	0.927	0.073
State self-kindness	0.825	0.736	0.927	–	–	–
State common humanity	0.847	0.865	0.919	–	–	–
State mindfulness	0.780	0.649	0.866	–	–	–
State self-judgment	0.772	0.444	0.919	–	–	–
State isolation	0.786	0.725	0.921	–	–	–
State over-identification	0.756	0.637	0.853	–	–	–
**Study 2 posttest**						
State self-compassion	0.938	0.974	0.956	0.840	0.879	0.121
State self-kindness	0.889	0.795	0.867	–	–	–
State common humanity	0.879	0.880	0.885	–	–	–
State mindfulness	0.823	0.636	0.830	–	–	–
State self-judgment	0.833	0.475	0.859	–	–	–
State isolation	0.871	0.763	0.860	–	–	–
State over-identification	0.801	0.625	0.822	–	–	–

*α, Cronbach’s alpha; CR, McDonald’s composite reliability; ω, omega; ω_H_, omega hierarchical; GF, reliable variance explained by the global factor; SF, reliable variance explained by the specific factors.*

*Items of the state self-judgment, isolation, and over-identification subscales were reverse-coded.*

#### Tests of Measurement Invariance

We tested the measurement invariance of the bifactor ESEM representation of the SSCS-J across sex groups (Table 4). The configural model had sufficient fit and the addition of each set of equality constraints on the factor loadings (i.e., weak), item thresholds (i.e., strong), item uniquenesses (i.e., strict), the latent variance–covariance matrix (i.e., latent variance–covariance invariance), and latent means (latent mean invariance) invariance showed that: (1) the CFI, TLI, and RMSEA indicated excellent fit to the data on all levels; (2) ΔCFI and ΔTLI were never above 0.010 with the highest being 0.006 for CFI and 0.05 for TLI; and (3) the ΔRMSEA never showed an increase of 0.015 or greater with the highest being 0.005. All these findings suggest that the SSCS-J functions the same way among Japanese men and women.

**TABLE 4:** Tests of measurement invariance of the bifactor ESEM of the Japanese version of the State Self-Compassion Scale across sexes in Study 1.

Models	χ^2^	df	CFI	TLI	RMSEA [90% CI]	Δχ^2^	Δdf	ΔCFI	ΔTLI	ΔRMSEA
Configural	165.681*	96	0.991	0.972	0.049 [0.036, 0.062]					
Weak	292.935*	173	0.985	0.974	0.048 [0.039, 0.058]	146.798*	173	−0.006	0.002	−0.001
Strong	349.656*	220	0.984	0.978	0.044 [0.036, 0.053]	73.417*	47	−0.001	0.004	−0.004
Strick	398.686*	238	0.980	0.974	0.048 [0.039, 0.056]	48.688*	18	−0.004	−0.004	0.004
Latent variance–covariance	449.838*	266	0.977	0.974	0.048 [0.040, 0.056]	82.957*	28	−0.003	0.000	0.000
Laten mean	497.971*	177	0.972	0.969	0.053 [0.045, 0.060]	30.868*	7	−0.005	−0.005	0.005

*p < 0.05.

*CFA, confirmatory factor analysis; ESEM, exploratory structural equation modeling; CFI, comparative fit index; TLI, Tucker–Lewis index; RMSEA, root-mean square error of approximation; CI, confidence interval.*

#### Construct Validity of the Japanese Version of the State Self-Compassion Scale

We calculated Pearson’s product-moment correlations between state self-compassion and the other study variables (Table 5). Most noteworthy are the correlations between the state and trait self-compassion which were strong yet not overly high, suggesting that the SSCS-J and the SCS-J would measure the different aspects of self-compassion (i.e., state versus trait levels). State self-compassion was associated with higher levels of positive affect and controllability appraisal, and lower levels of negative affect, threat appraisal, as well as fear and negative beliefs about self-compassion. These associations were similar to the relationships between trait self-compassion and these variables (see Supplementary Table 4, for more). In summary, these results support the construct validity of the SSCS-J.

**TABLE 5:** Descriptive statistics of and Pearson’s correlations between state self-compassion and other study variables in Study 1.

	*M*	SD	1	2	3	4	5	6	7
1. State self-compassion	2.99	0.66	–						
2. State self-kindness	2.83	0.93	0.650**	–					
3. State common humanity	3.09	0.98	0.474**	0.266**	–				
4. State mindfulness	2.93	0.89	0.726**	0.534**	0.321**	–			
5. State self-judgment	3.12	0.98	0.784**	0.451**	0.102**	0.393**	–		
6. State isolation	3.06	1.10	0.750**	0.241**	0.186**	0.386**	0.605**	–	
7. State over-identification	2.91	0.92	0.708**	0.221**	0.064	0.399**	0.639**	0.573**	–
8. Controllability appraisal	2.56	0.97	0.586**	0.414**	0.241**	0.558**	0.423**	0.395**	0.384**
9. Threat appraisal	3.72	1.03	−0.260**	–0.003	–0.069	−0.125**	−0.261**	−0.316**	−0.264**
10. Importance appraisal	3.81	1.00	0.021	0.099*	0.095**	0.123**	–0.073	–0.060	–0.080
11. Positive affect	2.41	0.85	0.256**	0.311**	0.189**	0.357**	0.090*	0.091*	0.038
12. Negative affect	3.37	1.00	−0.476**	−0.141**	–0.018	−0.250**	−0.503**	−0.475**	−0.541**
13. Demerits of self-compassion	2.67	1.04	−0.440**	−0.259**	–0.011	−0.187**	−0.511**	−0.419**	−0.394**
14. Miserable with self-compassion	2.37	1.01	−0.581**	−0.373**	−0.127**	−0.323**	−0.593**	−0.502**	−0.446**
15. Negative beliefs about self-compassion	2.70	1.02	−0.360**	−0.178**	0.078	−0.158**	−0.454**	−0.366**	−0.379**
16. Trait self-compassion	2.98	0.58	0.714**	0.489**	0.278**	0.547**	0.600**	0.513**	0.501**
17. Trait self-kindness	2.93	0.84	0.476**	0.600**	0.270**	0.443**	0.333**	0.169**	0.173**
18. Trait common humanity	3.05	0.85	0.316**	0.302**	0.477**	0.308**	0.104*	0.072	0.053
19. Trait mindfulness	3.15	0.81	0.482**	0.429**	0.231**	0.514**	0.334**	0.237**	0.262**
20. Trait self-judgment	3.10	0.82	0.497**	0.276**	0.028	0.248**	0.578**	0.461**	0.423**
21. Trait isolation	3.15	1.04	0.573**	0.204**	0.113**	0.354**	0.541**	0.606**	0.496**
22. Trait over-identification	2.50	0.92	0.471**	0.127**	0.030	0.327**	0.442**	0.441**	0.550**

*p < 0.05, **p < 0.01.

*Items of the state/trait self-judgment, isolation, and over-identification subscales were reverse-coded.*

#### Unique Relations of State and Trait Self-Compassion to Situational and Dispositional Variables

We estimated a path model in which state and trait self-compassion were specified to predict the other variables (Table 6). As expected, state self-compassion was more strongly associated with situational variables, such as, high controllability appraisal, low threat appraisal, and low negative affect in comparison to trait self-compassion. State self-compassion was not significantly related to the disposition to hold negative beliefs about self-compassion as anticipated. In contrast, state and trait levels of self-compassion were associated with high positive affect and low fear of self-compassion to the same degree.

**TABLE 6:** Path model of state and trait self-compassion in relation to cognitive appraisals, affect, fear of self-compassion, and negative beliefs about self-compassion.

Predictors	*B*	SE	β	*B*	SE	β	*B*	SE	β	*B*	SE	β
				
	Controllability appraisal			Threat appraisal			Importance appraisal			Positive affect		
State self-compassion	0.622**	0.068	0.424	−0.403**	0.088	−0.259	0.016	0.089	0.011	0.149*	0.072	0.115
Trait self-compassion	0.377**	0.077	0.227	−0.002	0.100	−0.001	0.024	0.101	0.014	0.289**	0.082	0.198
Proportion of explained variance	*R*^2^ = 0.368**			*R*^2^ = 0.067**			*R*^2^ = 0.001			*R*^2^ = 0.085**		

	**Negative affect**			**Demerit of self-compassion**			**Miserable with self-compassion**			**Negative beliefs about self-compassion**		

State self-compassion	−0.580**	0.078	−0.382	−0.334**	0.08	−0.213	−0.499**	0.07	−0.325	−0.157	0.081	−0.102
Trait self-compassion	−0.226*	0.088	−0.131	−0.566**	0.091	−0.318	−0.625**	0.079	−0.359	−0.631**	0.092	−0.362
Proportion of explained variance	*R*^2^ = 0.235**			*R*^2^ = 0.243**			*R*^2^ = 0.401**			*R*^2^ = 0.194**		

*p < 0.05, **p < 0.01.

*B, unstandardized regression coefficient, SE, standard error associated with the B value; β, standardized regression coefficient.*

#### Evaluation of the Short Version of the Japanese Version of the State Self-Compassion Scale

We finally tested whether the short version of the SSCS-J would have an adequate model fit and reliability. Following the previous research (Neff et al., 2021), we used six items to conduct CFA (see Supplementary Table 1). The one-factor CFA solution reasonably fit to the data (CFI = 0.941, TLI = 0.901, RMSEA = 0.098 [90% CI 0.076, 0.122]). Note that RMSEA did not reach a cut-off point, but this might be due to the small degree of freedom and thus does not necessarily indicate poor model fit (Kenny et al., 2015; see also Neff et al., 2021, for a similar result). Factor loadings were statistically significant and ranged from 0.105 to 0.757 (*M* = 0.488). Although Cronbach’s α coefficient (α = 0.604) was slightly lower than the typical thresholds, this short form correlated highly with the SSCS-J, *r* = 0.90, *p* < 0.001.

## Study 2

In Study 2, we aimed to cross-validate the bifactor ESEM representation of the SSCS-J and examined whether the experimental manipulation of self-compassion increased state self-compassion. Following Neff et al. (2021, Study 2), we adopted the self-compassionate mindstate induction (SCMI), which instructed participants to reflect on their painful experience from the perspective of self-compassion. We further extended Neff et al. (2021) by investigating the influence of SCMI on trait self-compassion.

### Method

#### Participants

We recruited participants in Japan through a crowdsourcing company (Lancers Inc.). A total of 496 participants agreed to participate in the online experiment on Qualtrics and these participants were paid 200 yen (approximately 2 United States dollars). Informed consent was obtained electronically. We excluded the data of 22 participants who either failed the attention check item (*n* = 11), left a blank on the writing task (*n* = 1), or reported that they could not recall their personal suffering (*n* = 10). The final sample of 474 participants (230 men, 244 women; *M*_age_ = 42.0, *SD* = 9.6) were randomly assigned to either the SCMI condition (*n* = 223) or the control condition (*n* = 251).

#### Procedure

First, participants were asked to recall a personal experience that was still causing them pain or suffering and to indicate how well they could recall such event (1 = *I could not recall it at all*, 2 = *I could recall it somewhat*, 3 = *I could recall it*, 4 = *I could recall it sufficiently*) and how much suffering they have been experiencing (i.e., “This event makes me suffer”) on a 5-point scale (1 = *not at all true for me*, 5 = *very true for me*). They also completed the pretest measure of state self-compassion.

Next, participants were randomly assigned to the SCMI or the control condition. Participants in the SCMI condition wrote about their personal suffering in a compassionate way (Neff et al., 2021). Specifically, they were instructed to accept and validate any thoughts and feelings about their painful situation (i.e., mindfulness), to recognize how their experience could be shared by others (i.e., common humanity), and to give care and understanding to themselves (i.e., self-kindness). Subsequently, they were asked to reflect on their writing. Participants in the control condition described their personal suffering in an objective manner (Neff et al., 2021). Specifically, they were instructed to elaborate on what happened, who was involved, and what words were spoken in the situation, and then to reflect on their writing (see Supplementary Appendix 1, for the Japanese instructions of the SCMI and control writing).

Finally, participants completed the posttest measures of state and trait self-compassion. Participants were then debriefed and thanked for their participation.

The instructions for both SCMI and control conditions were translated though the back-translation method. This research was approved by the Institutional Review Board of the affiliated university of the first author.

#### Measures

##### State Self-Compassion at Pretest and Posttest

State self-compassion at pretest and posttest were assessed with the SSCS-J. As in Study 1, participants were instructed to indicate the degree to which each item described their responses toward their recalled situation on a 5-point scale ranging from 1 (*not at all true for me*) to 5 (*very true for me*).

##### Self-Compassionate Reactions in General

Self-compassionate reactions in general (i.e., trait self-compassion) were assessed with the Japanese version of the Self-Compassionate Reactions Inventory (SCRI; Leary et al., 2011; Miyagawa and Taniguchi, 2016). The SCRI is an indicator of trait self-compassion because this scale measures self-compassion in broad situations (Leary et al., 2011) and strongly correlates with the SCS (*r* = 0.62, Miyagawa and Taniguchi, 2016). Given that the items of the SCS are highly similar to those of the SSCS, we measured trait self-compassion using the SCRI.

Participants were given eight hypothetical negative situations (e.g., “You failed to achieve a goal that was very important to you”) and for each, they choose two responses that they would likely take out of four response options. Two represented self-compassion (e.g., “I would think that these kinds of things happen to everyone”) and the other two were filler items. For each situation, participants received a score from 0 to 2. Following previous research (Leary et al., 2011; Miyagawa and Taniguchi, 2016), we calculated the sum of the self-compassionate reactions over the 8 situations with scores ranging from 0 to 16 (α = 0.903).

### Data Analyses

To test the factor structure of the SSCS-J, we followed the procedure of Study 1. Next, we confirmed no significant differences in suffering and the number of characters in the writing intervention between conditions using independent *t*-tests. Subsequently, following Neff et al. (2021, Study 2), we examined whether the SCMI increased state self-compassion relative to the control condition using 2 (Time: pretest vs. posttest) × 2 (Condition: the SCMI vs. the control) repeated-measures ANOVAs for state self-compassion. We used Cohen’s (1988) interpretations of partial eta squared: 0.01 as small, 0.06 as medium, and 0.14 and above as large. Finally, we tested whether participants in the SCMI condition reported higher self-compassionate reactions in general by using an independent *t*-test between conditions.

### Results and Brief Discussion

#### Cross-Validation of the Japanese Version of the State Self-Compassion Scale

Table 7 represents model fit results for the SSCS-J at pretest and posttest. Consistent with Study 1, we found that the bifactor ESEM representation of the SSCS-J had better fit than the bifactor and two-bifactor CFA solutions, while the differences in fit between the bifactor and two-bifactor ESEM models were negligible. We, then, examined the parameter estimates of the bifactor ESEM model at pretest and posttest (see Supplementary Tables 5, 6). The G-factor (self-compassion) was well-defined as shown in significant standard factor loadings at pretest (λ = 0.293–0.734, *M* = 0.577) and posttest (λ = 0.494–0.885, *M* = 0.695). Except the self-judgment subscale, S-factors were also well-defined with the significant loadings on the intended factors across the measurement times (see Supplementary Tables 5, 6, for more). In contrast, the two-bifactor ESEM model was characterized by identification issues and inadequate parameter estimates (e.g., inflated standard errors, invalid standardized factor loadings, large correlations among the two G-factors), arguing against the need to incorporate a second global factor.

**TABLE 7:** Goodness-of-fit indices for the estimated solution for the Japanese version of the State Self-Compassion Scale in Study 2.

Models	χ^2^	df	CFI	TLI	RMSEA [90% CI]
**Pretest**					
One factor CFA	3055.129*	135	0.663	0.618	0.214 [0.207, 0.220]
Two-factor CFA	2147.804*	134	0.768	0.735	0.178 [0.171, 0.185]
Two-factor ESEM	1627.382*	118	0.826	0.774	0.164 [0.157, 0.171]
Six-factor CFA	458.321*	120	0.961	0.950	0.077 [0.070, 0.085]
Six-factor ESEM	192.379*	60	0.985	0.961	0.068 [0.058, 0.079]
Bifactor CFA	749.172*	117	0.927	0.905	0.107 [0.100, 0.114]
Bifactor ESEM	98.143*	48	0.994	0.982	0.047 [0.034, 0.060]
Two-bifactor CFA	611.812*	116	0.943	0.925	0.095 [0.088, 0.102]
Two-bifactor ESEM^a^	55.678*	41	0.998	0.994	0.027 [0.000, 0.044]
**Posttest**					
One factor CFA	3114.306*	135	0.831	0.809	0.216 [0.209, 0.222]
Two-factor CFA	2191.378*	134	0.884	0.867	0.180 [0.173, 0.187]
Two-factor ESEM	1846.178*	118	0.902	0.873	0.176 [0.169, 0.183]
Six-factor CFA	457.241*	120	0.981	0.976	0.077 [0.070, 0.085]
Six-factor ESEM	157.089*	60	0.995	0.986	0.058 [0.047, 0.070]
Bifactor CFA	646.855*	117	0.970	0.961	0.098 [0.090, 0.105]
Bifactor ESEM	78.219*	48	0.998	0.995	0.036 [0.021, 0.051]
Two-bifactor CFA^a^	469.346*	116	0.980	0.974	0.080 [0.073, 0.088]
Two-bifactor ESEM^a^	66.737*	41	0.999	0.995	0.036 [0.019, 0.052]

*p < 0.05.

*CFI, comparative fit index; TLI, Tucker–Lewis index; RMSEA, root-mean square error of approximation; CI, confidence interval.*

*^a^The residual covariance matrix was not positive defined.*

The reliability of the bifactor ESEM model was sufficient at both pretest and posttest (Table 3). Specifically, except the self-judgment subscale, the model-based reliabilities were higher than the suggested cutoff-point (CR > 0.50). As in Study 1, the majority of the variance in the total scores was explained by the G-factor at pretest and posttest (93 and 88%, respectively). These results confirmed the cross-validation of the bifactor ESEM representation of the SSCS-J.

We also examined the model fit and reliability of the short six-item version of the SSCS-J. One-factor CFA showed the acceptable fit at pretest, CFI = 0.918, TLI = 0.864, RMSEA = 0.133 [90% CI 0.108, 0.160], and at posttest, CFI = 0.971, TLI = 0.952, RMSEA = 0.117 [90% CI 0.092, 0.144]. Factor loadings ranged from 0.435 to 0.692 (*M* = 0.580) at pretest and from 0.539 to 0.752 (*M* = 0.680) at posttest. Cronbach’s α coefficients at pretest (α = 0.709) and posttest (α = 0.808) were acceptable.

#### Preliminary Analyses

Independent *t*-tests showed no significant difference in suffering at pretest between the SCMI (*M* = 4.44, *SD* = 0.72) and control conditions (*M* = 4.48, *SD* = 0.72), *t*(472) = 0.65, *p* = 0.515, *d* = 0.06. Additionally, the SCMI condition (*M* = 234.40, *SD* = 111.61) and the control condition (*M* = 219.41, *SD* = 126.33) did not significantly differ in the number of words of the writing task, *t*(472) = 1.36, *p* = 0.174, *d* = 0.13.

#### Change in State Self-Compassion in the Self-Compassionate Mindstate Induction and Control Conditions

Table 8 represents the descriptive statistics of state self-compassion and its subcomponents at pretest and posttest between conditions. In the SCMI condition, the scores of state self-compassion appeared to increase from pretest and posttest, whereas this tendency was not evident in the control condition. We tested whether participants in the SCMI condition experienced greater changes in state self-compassion by conducting the 2 (Time: pretest vs. posttest) × 2 (Condition: the SCMI vs. the control) repeated-measures ANOVAs with Time as a within-subject factor and Condition as a between-subjects factor (Table 9). We found a significant main effect of Condition as well as a significant main effect of Time on each facet of the SSCS-J. As expected, these main effects were qualified by significant Time × Condition interactions with medium to large effect sizes.

**TABLE 8:** Descriptive statistics of the State Self-Compassion Scale at pretest and posttest between conditions.

	SCMI condition				Control condition			
	Pretest		Posttest		Pretest		Posttest	
	*M*	SD	*M*	SD	*M*	SD	*M*	SD
State self-compassion	2.83	0.66	3.61	0.67	2.81	0.70	2.88	0.76
State self-kindness	2.88	0.91	3.75	0.76	2.81	0.91	2.87	0.97
State common humanity	2.98	1.04	3.71	0.90	3.04	1.03	3.00	1.07
State mindfulness	2.85	0.90	3.54	0.78	2.78	0.91	2.87	0.91
State self-judgment	2.90	0.93	3.71	0.90	2.96	0.92	3.04	1.01
State isolation	2.70	1.03	3.53	1.02	2.64	1.03	2.75	1.14
State over-identification	2.66	0.88	3.44	0.83	2.65	0.93	2.76	0.96
State self-compassion short-form	2.71	0.72	3.55	0.68	2.71	0.72	2.80	0.78

*Items of the state self-judgment, isolation, and over-identification subscales were reverse-coded so that the higher scores indicate the absence of these types of uncompassionate self-responding.*

**TABLE 9:** Statistics for the repeated-measures analyses of variance for SSCS-J.

	Condition				Time				Condition by time			
	*F*	dfs	*P*	Partial eta^2^	*F*	dfs	*p*	Partial eta^2^	*F*	dfs	*p*	Partial eta^2^
State self-compassion	40.47	1, 472	<0.001	0.079	250.80	1, 472	<0.001	0.347	174.08	1, 472	<0.001	0.269
State self-kindness	40.86	1, 472	<0.001	0.080	167.15	1, 472	<0.001	0.262	126.94	1, 472	<0.001	0.212
State common humanity	14.35	1, 472	<0.001	0.029	86.00	1, 472	<0.001	0.154	103.82	1, 472	<0.001	0.180
State mindfulness	25.78	1, 472	<0.001	0.052	125.89	1, 472	<0.001	0.211	70.89	1, 472	<0.001	0.131
State self-judgment	15.52	1, 472	<0.001	0.032	132.10	1, 472	<0.001	0.219	89.81	1, 472	<0.001	0.160
State isolation	22.18	1, 472	<0.001	0.045	144.37	1, 472	<0.001	0.234	81.42	1, 472	<0.001	0.147
State over-identification	21.64	1, 472	<0.001	0.044	138.19	1, 472	<0.001	0.226	78.67	1, 472	<0.001	0.143
State self-compassion short-form	39.89	1, 472	<0.001	0.078	229.70	1, 472	<0.001	0.327	152.72	1, 472	<0.001	0.244

*Items of the state self-judgment, isolation, and over-identification subscales were reverse-coded so that the higher scores indicate the absence of these types of uncompassionate self-responding.*

We examined whether the pre-to-post changes in state self-compassion were significant for the SCMI condition. Simple main effect analyses with Bonferroni correction showed significant increases with large effect sizes in the total score as well as each subscale of the SSCS-J in the SCMI conditions, *F*s = 178.84–397.89, *p*s < 0.001, η_p_^2^ = 0.275–0.457. For the control condition, the pre-to-post changes were non-significant for overall state self-compassion, self-kindness, common humanity, and reduced self-judgment, *F*s = 0.45–3.71, *p*s = 0.055–0.505, η_p_^2^ = 0.001—0.008. On the other hand, the pre-to-post changes in state self-compassion short-form, mindfulness, reduced isolation, and reduced over-identification were significant, *F*s = 4.16–4.76, *p*s = 0.030–0.042, η_p_^2^ = 0.009—0.010. Note, however, that these changes were relatively small in effect sizes and compared with the SCMI condition.

Consistent with Neff et al. (2021), the results suggest that the SCMI effectively promotes state self-compassion and its effects are considered large. Whereas the control writing increases several aspects of state self-compassion, these effects are small. Simply writing about negative events in an objective fashion can create some psychological distance from negative events (Kross et al., 2005), which might help people gain greater perspective about the situation and alleviate stress. However, considering the effect sizes of the SCMI and control conditions, writing in a self-compassionate way seems to be more effective for boosting all dimensions of state self-compassion.

#### The Effect of the Self-Compassionate Mindstate Induction on Self-Compassionate Reactions in General

We examined whether the effect of SCMI is specific to state self-compassion or generalized to self-compassion in general. We did not find a significant difference between SCMI (*M* = 8.31, *SD* = 4.59) and control conditions (*M* = 7.64, *SD* = 4.71), *t*(472) = 1.57, *p* = 0.117, *d* = 0.14. Therefore, writing about a specific event in a self-compassionate way does not appear to immediately influence self-compassion in general.

In sum, we cross-validated the bifactor ESEM representation of the SSCS-J and found that state self-compassion in a situation significantly increased after the experimental manipulation of self-compassion in the situation. However, such manipulation did not affect the trait levels of self-compassion.

## General Discussion

We examined whether the proposed bifactor ESEM representation of state self-compassion (Neff et al., 2021) would be replicated in Japan. Although there is a debate on whether self-compassion should include both types of self-responding or if it should be treated as two separate factors (Muris et al., 2016, 2019; Neff et al., 2018, 2019), our results supported the former stance. Across two studies, in the bifactor ESEM model, we found that the self-compassion G-factor accounted for the large proportion of the variance in the total score, which justified the use of the total score as an index of state self-compassion (Neff et al., 2021). In contrast, even though the two-bifactor ESEM model (which incorporated two global factors representing compassionate and uncompassionate self-responding) demonstrated adequate model fit, most items did not load significantly on their intended G-factors. Therefore, in line with Neff et al. (2021), we did not find evidence for separating the types of self-responding as two G-factors. In sum, the bifactor ESEM representation of the SSCS-J was superior to all alternative solutions. Given that the reliability of the total score was also high, we recommend using the total score of 18 items rather than two separate scores of compassionate and uncompassionate self-responding.

Across the two studies, the results suggest that the bifactor ESEM representation of state self-compassion had an excellent fit and incorporated a well-defined and reliable self-compassion G-factor. In this model, with the exception of the self-judgment and over-identification S-factors, the other S-factors were also well-defined. This, however, should not be of concern given that in bifactor operationalizations a well-defined G-factor only needs to be accompanied by some well-defined S-factors (Morin et al., 2020). As such, observing weakly defined S-factors in a bifactor solution simply suggests that the items used to assess the specific component provide a clearer reflection of global levels of self-compassion than that of the specific component. Importantly, these results converge with the previous findings in the United States (Neff et al., 2021) and the theory that self-compassion is a balanced system between three compassionate self-responding (i.e., self-kindness, common humanity, and mindfulness) and three reduced uncompassionate self-responding (i.e., self-judgment, isolation, and over-identification; Neff, 2003; Neff et al., 2018, 2019).

Similar to the previous findings (Zessin et al., 2015; Neff et al., 2018, 2021; Sirois et al., 2019), state self-compassion was associated with higher positive affect and lower negative affect. Self-compassion functions as an emotion regulation strategy (Sirois et al., 2019) so that people with a self-compassionate mindset in a painful situation may keep their emotions in balance, be able to view their experience within a larger context, and access their inner resources to better cope with difficulty (Neff, 2003; Neff et al., 2018, 2019).

We extended the previous findings of this state measure (Neff et al., 2021) by testing its measurement invariance across sex groups in Study 1 (Meredith, 1993; Marsh et al., 2014; Tóth-Király and Neff, 2021). Similar to the trait self-compassion (Tóth-Király and Neff, 2021), we found the support for the equal factor structure (i.e., configural invariance), factor loading (i.e., weak invariance), item thresholds (i.e., strong invariance), and item uniqueness (i.e., strict invariance), as well as support for the equality of latent variances-covariances and latent factor means. Taken as a whole, the SSCS-J is an equivalent measure for men and women in Japan.

We provided further support for the construct validity of the SSCS-J by examining the relations of state self-compassion to trait self-compassion, cognitive appraisals, fear of self-compassion, and negative beliefs about self-compassion. State and trait self-compassion and their six components were significantly correlated with each other. Furthermore, state self-compassion correlated with a higher perception of controllability and lower threat appraisal of the situation, whereas it did not relate to the importance appraisal. These results were in line with the previous findings that trait self-compassion relates to better cognitive appraisals of a stressful event (Chishima et al., 2018; Miyagawa and Taniguchi, 2018).

Despite the conceptual overlap between state and trait self-compassion, we also found evidence that these two variables may measure different aspects of self-compassion. Indeed, our path model in Study 1 suggests that the relations of state self-compassion to cognitive appraisals of the situation and negative affect were stronger than the associations between trait self-compassion and these variables. This result highlights the unique contribution of state self-compassion compared to its trait counterpart. Regardless of the level of trait self-compassion, people who activate state self-compassion in challenging moments might have a greater capacity to appraise the situation as more controllable and less threatening. By focusing on state self-compassion, we can gain greater insight into how self-compassion functions within given contexts.

Additionally, the experimental manipulation of self-compassion in response to a difficult situation was effective for boosting state self-compassion but not for increasing trait self-compassion. This is likely because the development of trait self-compassion requires more time and practice through longer and more intensive interventions, such as the 8-week Mindful Self-Compassion program (Germer and Neff, 2019). Therefore, state and trait self-compassion are distinct and state self-compassion appears to be more responsive to a mindstate induction focused on a specific situation.

Our studies identified two variables that could undermine state self-compassion (Gilbert et al., 2011; Chwyl et al., 2020). Fear of self-compassion and negative beliefs about self-compassion were negatively correlated with state self-compassion. Importantly, the path model in Study 1 implies that fear of self-compassion may be more strongly related to state self-compassion. Thus, compared with misbeliefs about self-compassion, fear of self-compassion could play a larger role in hindering a person’s application of self-compassion in moments of difficulty. This result may also reflect emotional backdraft: the distress and uneasiness people feel when they start to foster self-compassion (Germer and Neff, 2019). Importantly, emotional backdraft is a common experience for people in both clinical and non-clinical settings and is considered a valuable part of developing self-compassion (Germer and Neff, 2019). Our results suggest that relieving the discomfort of backdraft may have a greater effect on one’s ability to be self-compassionate than attempting to improve negative beliefs about self-compassion (Chwyl et al., 2020). For example, to increase self-compassion, we could expose people to grounding practices for regulating one’s attention and soothing the nervous system in response to the emotional distress of backdraft. This could provide individuals with the emotional safety to approach the practice of self-compassion and receive the benefits of being self-compassionate in moments of suffering.

### Limitations and Future Directions

We validated SSCS-J by examining its associations with other related constructs; however, as with all correlational studies, we cannot claim causality or directionality of these associations. Activating state self-compassion in a painful situation might promote greater appraisal of controllability and lower threat appraisal (e.g., Chishima et al., 2018; Miyagawa and Taniguchi, 2018). Conversely, people might increase state self-compassion because a situation is perceived as more controllable and less threatening. A longitudinal or experimental study is necessary to clarify the directionality of state self-compassion and cognitive appraisals of the situation.

Second, it is unclear what situational variables may moderate state self-compassion. Even when people are at the same levels of trait self-compassion, there may be within-person variability in how easily they could activate state self-compassion. For example, some self-compassionate people may have an easier time being self-compassionate within certain contexts (e.g., academic failure), and may struggle to be self-compassionate in other situations (e.g., relationship conflicts). One interesting future direction is to examine the within-person variability of self-compassion that is influenced by domains or characteristics of situations.

Third, we did not examine the associations between state self-compassion and a diverse range of mental health, and psychological well-being indicators. Given that the robust and strong links between trait self-compassion and these variables (MacBeth and Gumley, 2012; Zessin et al., 2015), we expect that state self-compassion may help people maintain higher levels of mental health and psychological well-being in a painful situation. Future longitudinal research is needed to directly test this prediction. Additionally, we acknowledge that social desirability may affect participants’ responses to the SSCS-J. Although we expect that this possibility may be low because the SCS, from which the SSCS was created, showed no association with social desirability (Neff, 2003; Barnard and Curry, 2011), future research should clarify whether state self-compassion is not related to social desirability.

Fourth, although we showed that the Japanese version of the SCMI increased state self-compassion, further research on this manipulation is necessarily. For example, research showed that simply writing compassionate messages to the self (i.e., self-kindness) increased self-improvement motivation after failure (Breines and Chen, 2012). An interesting direction is to examine whether the common humanity and mindfulness components of the SCMI would magnify the effect of self-kindness on self-improvement motivation.

Fifth, we relied on a sample of Japanese participants which, naturally, limits the generalizability of our findings to other groups. Replications thus should be made in other cultural and linguistic contexts.

Despite these limitations, our study contributes to the literature by providing a new scale to measure state self-compassion in Japan and demonstrating the construct validity of this scale. In line with Neff et al. (2021), our study suggests that the SSCS-J has a bifactor ESEM representation including one G-factor (self-compassion) and six S-factors (six subscales) that correspond to the theoretical assumption of the psychological balance between compassionate self-responding and reduced uncompassionate self-responding (Neff, 2003; Neff et al., 2018, 2019).

## Data Availability Statement

The datasets for Studies 1 and 2 are available on request to the corresponding author.

## Ethics Statement

The studies involving human participants were reviewed and approved by the Otemon Gakuin University. Written informed consent for participation was not required for this study in accordance with the national legislation and the institutional requirements.

## Author Contributions

YM, MK, JT, and YN contributed to conception and design of the study. YM collected the data. YM and IT-K performed the statistical analysis. YM wrote the first draft of the manuscript in consultation with JT and YN. IT-K and MK wrote sections of the manuscript. All authors read and approved the submitted version.

## Conflict of Interest

The authors declare that the research was conducted in the absence of any commercial or financial relationships that could be construed as a potential conflict of interest.

## Publisher’s Note

All claims expressed in this article are solely those of the authors and do not necessarily represent those of their affiliated organizations, or those of the publisher, the editors and the reviewers. Any product that may be evaluated in this article, or claim that may be made by its manufacturer, is not guaranteed or endorsed by the publisher.

## References

[B1] Asano K. Tsuchiya M. Ishimura I. Lin S. Matsumoto Y. Miyata H. (2017). The development of fears of compassion scale Japanese version. *PLoS One* 12:e0185574. 10.1371/journal.pone.0185574 29023461 PMC5638239

[B2] Barnard L. K. Curry J. F. (2011). Self-compassion: conceptualizations, correlates, & interventions. *Rev. Gen. Psychol.* 15 289–303. 10.1037/a0025754

[B3] Breines J. G. Chen S. (2012). Self-compassion increases self-improvement motivation. *Pers. Soc. Psychol. Bull.* 38 1133–1143. 10.1177/0146167212445599 22645164

[B4] Browne M. (2001). An overview of analytic rotation in exploratory factor analysis. *Multivariate Behav. Res.* 36 111–150. 10.1207/S15327906MBR3601_05

[B5] Brunner M. Nagy G. Wilhelm O. (2012). A tutorial on hierarchically structured constructs. *J. Personal.* 80 796–846. 10.1111/j.1467-6494.2011.00749.x 22091867

[B6] Chen F. F. (2007). Sensitivity of goodness of fit indexes to lack of measurement invariance. *Struct. Equ. Modeling* 14 464–504. 10.1080/10705510701301834

[B7] Cheung G. W. Rensvold R. B. (2002). Evaluating goodness-of-fit indexes for testing measurement invariance. *Struct. Equ. Modeling* 9 233–255. 10.1207/S15328007SEM0902_5 33486653

[B8] Chishima Y. Mizuno M. Sugawara D. Miyagawa Y. (2018). The influence of self-compassion on cognitive appraisals and coping with stressful events. *Mindfulness* 9 1907–1915. 10.1007/s12671-018-0933-0

[B9] Chwyl C. Chen P. Zaki J. (2020). Beliefs about self-compassion: implications for coping and self-improvement. *Pers. Soc. Psychol. Bull.* 47 1327–1342. 10.1177/0146167220965303 33166205

[B10] Cohen J. (1988). *Statistical Power Analysis for the Behavioral Sciences*, 2nd Edn. Oxfordshire: Erlbaum.

[B11] Comrey A. L. Lee H. B. (2013). *A First Course in Factor Analysis.* East Sussex, UK: Psychology Press.

[B12] de Winter J. C. F. Dodou D. Wieringa P. A. (2009). Exploratory factor analysis with small sample sizes. *Multivariate Behav. Res.* 44 147–181. 10.1080/00273170902794206 26754265

[B13] Ewert C. Vater A. Schröder-Abé M. (2021). Self-compassion and coping: a meta-analysis. *Mindfulness* 12 1063–1077. 10.1007/s12671-020-01563-8

[B14] Finney S. J. DiStefano C. (2013). “Non-normal and categorical data in structural equation modeling,” in *Structural Equation Modeling: A Second Course*, 2nd Edn, eds Hancock G. R. Mueller R. O. (Charlotte, North Carolina: Information Age Publishing), 439–492.

[B15] Germer C. K. Neff K. D. (2019). *Teaching the Mindful Self-Compassion Program; A Guide for Professionals.* New York: The Guilford Press.

[B16] Gilbert P. McEwan K. Matos M. Rivis A. (2011). Fears of compassion: development of three self-report measures. *Psychol. Psychother.* 84 239–255. 10.1348/147608310X526511 22903867

[B17] Hu L. Bentler P. M. (1999). Cutoff criteria for fit indexes in covariance structure analysis: Conventional criteria versus new alternatives. *Struct. Equ. Modeling* 6 1–55. 10.1080/10705519909540118

[B18] Kato T. (2001). Interpersonal stress. *JPN. J. Educ. Psychol.* 49 295–304. 10.5926/jjep1953.49.3_295

[B19] Kawahito J. Otsuka Y. Kaida K. Nakata A. (2011). Reliability and validity of the Japanese version of 20-item Positive and Negative Affect Schedule. *Hiroshima Psychol. Res.* 11 225–240.

[B20] Kenny D. A. Kaniskan B. McCoach D. B. (2015). The performance of RMSEA in models with small degrees of freedom. *Sociol. Methods Res.* 44 486–507. 10.1177/0049124114543236

[B21] Kross E. Ayduk O. Mischel W. (2005). When asking “why” does not hurt: Distinguishing rumination from reflective processing of negative emotions. *Psychol. Sci.* 16 709–715. 10.1111/j.1467-9280.2005.01600.x 16137257

[B22] Lathren C. R. Rao S. S. Park J. Bluth K. (2021). Self-compassion and current close interpersonal relationships: a scoping literature review. *Mindfulness* 12 1078–1093. 10.1007/s12671-020-01566-5 PMC8932676 35309268

[B23] Leary M. R. Terry M. L. Allen A. B. Guadagno J. (2011). *Self-Compassionate Reactions Inventory.* Durham, NC: Duke University. (Unpublished manuscript).

[B24] MacBeth A. Gumley A. (2012). Exploring compassion: a meta-analysis of the association between self-compassion and psychopathology. *Clin. Psychol. Rev.* 32 545–552. 10.1016/j.cpr.2012.06.003 22796446

[B25] Marsh H. W. Hau K.-T. Grayson D. (2005). “Goodness of Fit in Structural Equation Models,” in *Contemporary Psychometrics: A Festschrift for Roderick P. McDonald*, eds Maydeu-Olivares A. McArdle J. J. (Mahwah, NJ: Lawrence Erlbaum Associates Publishers), 275–340.

[B26] Marsh H. W. Hau K. T. Wen Z. (2004). In search of golden rules: comment on hypothesis-testing approaches to setting cutoff values for fit indexes and dangers in overgeneralizing Hu and Bentler’s (1999) findings. *Struct. Equ. Modeling* 11 320–341. 10.1207/s15328007sem1103_2

[B27] Marsh H. W. Morin A. J. Parker P. D. Kaur G. (2014). Exploratory structural equation modeling: an integration of the best features of exploratory and confirmatory factor analysis. *Ann. Rev. Clin. Psychol.* 10 85–110. 10.1146/annurev-clinpsy-032813-153700 24313568

[B28] Meredith W. (1993). Measurement invariance, factor analysis and factorial invariance. *Psychometrika* 58 525–543. 10.1007/BF02294825

[B29] Millsap R. E. (2011). *Statistical Approaches to Measurement Invariance.* Oxfordshire: Taylor & Francis.

[B30] Miyagawa Y. Niiya Y. Taniguchi J. Morishita T. (2015). Development of the Japanese version of the Self-Compassion Scale (SCS-J). *Tezukayama Univ. Bull. Psychol.* 4 67–75.

[B31] Miyagawa Y. Taniguchi J. (2016). Development of the Japanese version of the Self-Compassionate Reactions Inventory. *JPN. J. Psychol.* 87 70–78. 10.4992/jjpsy.87.14220 27180515

[B32] Miyagawa Y. Taniguchi J. (2018). Investigating the influence of self-compassion on coping with job rejection. *JPN. J. Soc. Psychol.* 33 103–114.

[B33] Morin A. J. S. Arens A. K. Marsh H. W. (2016). A bifactor exploratory structural equation modeling framework for the identification of distinct sources of construct-relevant psychometric multidimensionality. *Struct. Equ. Modeling* 23 116–139. 10.1080/10705511.2014.961800

[B34] Morin A. J. S. Myers N. D. Lee S. (2020). “Modern factor analytic techniques: Bifactor models, exploratory structural equation modeling (ESEM) and bifactor-ESEM,” in *Handbook of Sport Psychology*, 4th Edn, eds Tenenbaum G. Eklund R. C. (Hoboken, New Jersey: Wiley), 1044–1073.

[B35] Muraven M. Baumeister R. F. (2000). Self-regulation and depletion of limited resources: does self-control resemble a muscle? *Psychol. Bull.* 126 247–259. 10.1037/0033-2909.126.2.247 10748642

[B36] Muris P. Otgaar H. Petrocchi N. (2016). Protection as the mirror image of psychopathology: Further critical notes on the Self-Compassion Scale. *Mindfulness* 7 787–790. 10.1007/s12671-016-0509-9

[B37] Muris P. Otgaar H. Pfattheicher S. (2019). Stripping the forest from the rotten trees: compassionate self-responding is a way of coping, but reduced uncompassionate self-responding mainly reflects psychopathology. *Mindfulness* 10 196–199. 10.1007/s12671-018-1030-0

[B38] Muthén L. K. Muthén B. O. (2015). *Mplus User’s Guide*, 7th Edn. Los Angeles, CA: Muthén & Muthén.

[B39] Neff K. D. (2003). The development and validation of a scale to measure self-compassion. *Self Identity* 2 223–250. 10.1080/15298860309027 26979311

[B40] Neff K. D. Long P. Knox M. C. Davidson O. Kuchar A. Costigan A. (2018). The forest and the trees: examining the association of self-compassion and its positive and negative components with psychological functioning. *Self. Identity* 17 627–645. 10.1080/15298868.2018.1436587

[B41] Neff K. D. Tóth-Király I. Knox M. C. Kuchar A. Davidson O. (2021). The development and validation of the State Self-Compassion Scale (Long- and Short Form). *Mindfulness* 12 121–140. 10.1007/s12671-020-01505-4

[B42] Neff K. D. Tóth-Király I. Yarnell L. M. Arimitsu K. Castilho P. Ghorbani N. (2019). Examining the factor structure of the Self-Compassion Scale in 20 diverse samples: support for use of a total score and six subscale scores. *Psychol. Assess.* 31 27–45. 10.1037/pas0000629 30124303

[B43] Perreira T. A. Morin A. J. Hebert M. Gillet N. Houle S. A. Berta W. (2018). The short form of the Workplace Affective Commitment Multidimensional Questionnaire (WACMQ-S): a bifactor-ESEM approach among healthcare professionals. *J. Vocat. Behav.* 106 62–83. 10.1016/j.jvb.2017.12.004

[B44] Reise S. P. (2012). The rediscovery of bifactor measurement models. *Multivariate Behav. Res.* 47 667–696. 10.1080/00273171.2012.715555 24049214 PMC3773879

[B45] Reise S. P. Bonifay W. E. Haviland M. G. (2013). Scoring and modeling psychological measures in the presence of multidimensionality. *J. Pers. Assess.* 95 129–140. 10.1080/00223891.2012.725437 23030794

[B46] Rodriguez A. Reise S. P. Haviland M. G. (2016). Applying bifactor statistical indices in the evaluation of psychological measures. *J. Pers. Assess.* 98 223–237. 10.1080/00223891.2015.1089249 26514921

[B47] Sirois F. M. Nauts S. Molnar D. S. (2019). Self-compassion and bedtime procrastination: an emotion regulation perspective. *Mindfulness* 10 434–445. 10.1007/s12671-018-0983-3

[B48] Tóth-Király I. Bőthe B. Orosz G. (2017). Exploratory structural equation modeling analysis of the Self-Compassion Scale. *Mindfulness* 8 881–892. 10.1007/s12671-016-0662-1 PMC5681952 29163325

[B49] Tóth-Király I. Neff K. D. (2021). Is self-compassion universal? Support for the measurement invariance of the Self-Compassion Scale across populations. *Assessment* 28 169–185. 10.1177/1073191120926232 32475146

[B50] Yu C. Y. (2002). *Evaluating Cutoff Criteria of Model Fit Indices for Latent Variable Models with Binary and Continuous Outcomes.* Ph.D.thesis, Los Angeles, CA: University of California.

[B51] Zessin U. Dickhäuser O. Garbade S. (2015). The relationship between self-compassion and wellbeing: a meta-analysis. *Appl. Psychol. Health Well Being* 7 340–364. 10.1111/aphw.12051 26311196

