# Supplementary material for: Development of the Japanese Version of the State Self-Compassion Scale (SSCS-J)
Source: Front Psychol. 2022 Jan 14;12:779318. doi: 10.3389/fpsyg.2021.779318 (PMC8795373; doi:10.3389/fpsyg.2021.779318)
Supplement: Supplementary file 1 [file Data_Sheet_1.docx]

Supplementary Online Materials for Development of the Japanese version of the State Self-Compassion Scale: A replication and extension of Neff et al. (2021)

**Table S1**

*The Japanese Version of the State Self-Compassion Scale*

| Item number | Subscale | Neff et al.'s (2021) original items and their Japanese translation |
| --- | --- | --- |
| 1 | SSK1 | I’m giving myself the caring and tenderness I need. |
|  |  | I am giving myself the kindness and love that I need (back-translation). |
|  |  | 自分自身に必要な優しさや愛情をそそいでいる |
| 7 | SSK2 | I am being kind to myself. |
|  |  | I am being kind to myself (back-translation). |
|  |  | 自分自身にやさしくしている |
| 13 | SSK3 | I’m being supportive toward myself. |
|  |  | I am being supportive of myself (back-translation). |
|  |  | 自分自身のこころに寄り添っている |
| 4 | SSJ1 | I’m being pretty tough on myself. |
|  |  | I am being overly critical about myself (back-translation). |
|  |  | 自分自身にひどく批判的になっている |
| 10 | SSJ2 | I’m being a bit cold-hearted towards myself. |
|  |  | I am treating myself coldly (back-translation). |
|  |  | 自分自身に冷たく接している |
| 16 | SSJ3 | I feel intolerant and impatient toward myself. |
|  |  | I am feeling intolerant and irritated about myself (back-translation). |
|  |  | 自分自身に寛容でいられず、イライラしている |
| 3 | SCH1 | I see my difficulties as part of life that everyone goes through. |
|  |  | I am thinking that my hardship is part of life that everybody goes through (back-translation). |
|  |  | 私が抱くつらさは誰もが経験する人生の一部分だとしてとらえている |
| 9 | SCH2 | I'm remembering that there are lots of others in the world feeling like I am. |
|  |  | I am recalling that many people in the world feel the same way as I do (back-translation). |
|  |  | 世の中の多くの人々が自分と同じように感じることがあるということを思い起こしている |
| 15 | SCH3 | I’m remembering that difficult feelings are shared by most people. |
|  |  | I am remembering that many people can feel similar distressing emotions (back-translation). |
|  |  | 多くの人々も同じようにつらい気持ちを感じるものだということを思い起こしている |
| 6 | SIS1 | I feel separate and cut off from the rest of the world. |
|  |  | I am feeling isolated and cut off from others in the world (back-translation). |
|  |  | 世の中の他の人々から切り離されて、孤立しているように感じている |
| 12 | SIS2 | I feel like I’m struggling more than others right now. |
|  |  | Right at this moment, I feel that I am struggling more than others (back-translation). |
|  |  | 今この瞬間、私は他の人々よりももがき苦しんでいるように感じている |
| 18 | SIS3 | I’m feeling all alone right now. |
|  |  | Right at this moment, I am feeling all alone (back-translation). |
|  |  | 今この瞬間、独りぼっちであるように感じている |
| 5 | SMI1 | I’m keeping my emotions in balanced perspective. |
|  |  | I am keeping a balance in my feelings (back-translation). |
|  |  | 自分の気持ちのバランスを保っている |
| 11 | SMI2 | I’m taking a balanced view of this painful situation. |
|  |  | I am keeping a balanced perspective of this painful situation (back-translation). |
|  |  | この痛ましい状況をバランスよく捉えている |
| 17 | SMI3 | I’m keeping things in perspective. |
|  |  | I am seeing things from a broader perspective (back-translation). |
|  |  | 物事を包括的に捉えている |
| 2 | SOI1 | I’m obsessing and fixating on everything that’s wrong. |
|  |  | I am obsessed with every negative aspect and am caught up in it (back-translation). |
|  |  | 悪いこと1つ1つに執着し、そのことにとらわれてしまっている |
| 8 | SOI2 | I’m getting carried away with my feelings. |
|  |  | I am being overwhelmed with my emotions (back-translation). |
|  |  | 自分の気持ちに圧倒されてしまっている |
| 14 | SOI3 | I’m blowing this painful incident out of proportion. |
|  |  | I am overreacting to this painful event (back-translation). |
|  |  | この痛ましい出来事に過剰に反応してしまっている |

*Note*. SSK = state self-kindness. SSJ = state self-judgment. SCH = state common humanity. SIS = state isolation. SMI = state mindfulness. SOI = state overidentification. Items of the state self-judgment, isolation, and overidentifications are reverse-coded. The Short form of this scale comprise the following items: No. 1, 2, 9, 12, 16, 17. The Japanese translation of the instruction is as follow: つらい気持ちや痛ましい気持ちを今この瞬間も経験している状況を1つ選んで、それを思い出してください。例えば、あなたが人生において直面している壁や、何らかの点で自らの至らなさを感じている状況です。その状況を思い起こしている時、あなたがご自身に対してどのように感じているのかをお尋ねします。各項目が今この瞬間のあなたにどの程度あてはまるのかを答えてください。

**Table S2**

*Standardized Factor Loadings for the Six-Factor CFA and ESEM Solution for the Japanese Version of the State Self-Compassion Scale in Study 1*

|  | CFA | ESEM | | | | | |
| --- | --- | --- | --- | --- | --- | --- | --- |
|  | Factor (λ) | SK (λ) | SJ (λ) | CH (λ) | IS (λ) | MI (λ) | OI (λ) |
| SSK1 | **.673**** | **.656**** | .174** | .020 | -.102* | .041 | -.043 |
| SSK2 | **.818**** | **.807**** | .024 | .000 | -.008 | .115** | -.027 |
| SSK3 | **.756**** | **.489**** | .110* | .102** | .033 | .272** | -.120** |
| SSJ1 | **.788**** | .007 | **.715**** | .037 | .117** | .060 | .086 |
| SSJ2 | **.721**** | .407** | **.329**** | .034 | .211** | -.234** | .186*** |
| SSJ3 | **.746**** | .155** | **.331**** | -.082 | .166** | .065 | .292** |
| SCH1 | **.725**** | .067 | -.049 | **.583**** | .191** | -.011 | -.011 |
| SCH2 | **.713**** | -.079** | .103** | **.877**** | -.152** | -.014 | -.020 |
| SCH3 | **.794**** | .019 | -.093* | **.760**** | .032 | .037 | -.012 |
| SIS1 | **.842**** | -.101** | .099** | -.005 | **.959**** | .041 | -.139** |
| SIS2 | **.694**** | -.039 | .023 | .121** | **.405**** | -.078* | .367** |
| SIS3 | **.842**** | .008 | .027 | -.019 | **.796**** | .093** | -.026 |
| SMI1 | **.801**** | .310** | -.024 | .068* | .087* | **.410**** | .156** |
| SMI2 | **.730**** | .120** | -.049 | .085** | .058 | **.637**** | .096* |
| SMI3 | **.639**** | .015 | .007 | -.005 | .039 | **.696**** | .052 |
| SOI1 | **.752**** | -.042 | .492** | .009 | .004 | .127** | **.316**** |
| SOI2 | **.560**** | -.017 | .133* | -.023 | .114* | -.060** | **.462**** |
| SOI3 | **.730**** | -.116** | -.009 | -.008 | .045 | .231** | **.757**** |

*Note*. **p* < .05; ***p* < .01; λ = standardized factor loading; CFA = confirmatory factor analysis; ESEM = exploratory structural equation modeling; SSK = state self-kindness. SSJ = state self-judgment. SCH = state common humanity. SIS = state isolation. SMI = state mindfulness. SOI = state overidentification. Target loadings are bolded. Items of the Self-Judgment, Isolation, and Over-Identification subscales were reverse-coded.

**Table S3**

*Standardized Factor Loadings for the Two-Bifactor ESEM Solution for the Japanese Version of the State Self-Compassion Scale in Study 1*

|  | CS (λ) | RUS (λ) | SK (λ) | SJ (λ) | CH (λ) | IS (λ) | MI (λ) | OI (λ) |
| --- | --- | --- | --- | --- | --- | --- | --- | --- |
| SSK1 | -.278** |  | **.552**** | .305** | .289** | -.027 | .118 | .044 |
| SSK2 | -.269 |  | **.685**** | .224** | .246** | .080* | .229** | .041 |
| SSK3 | .082 |  | **.684**** | .181** | .005 | .112** | .373** | -.058 |
| SSJ1 |  | -.169 | .199** | **.603**** | .048 | .247** | .141** | .394** |
| SSJ2 |  | -.036 | .462** | **.446**** | -.003 | .345** | -.073 | .327** |
| SSJ3 |  | .078 | .210** | **.568**** | -.128** | .390** | .202** | .208** |
| SCH1 | .263* |  | .187** | .047 | **.522**** | .250** | .155** | -.032 |
| SCH2 | .616** |  | .195** | .001 | **.526**** | -.031 | .164* | -.029 |
| SCH3 | .462** |  | .250** | -.135** | **.523**** | .098* | .198** | .035 |
| SIS1 |  | -.507** | .079* | .197** | .049 | **.763**** | .145** | .256** |
| SIS2 |  | .114 | -.060 | .353** | .180** | **.613**** | -.091 | .190* |
| SIS3 |  | -.219 | .145** | .245** | .027 | **.642**** | .213** | .209** |
| SMI1 | -.069 |  | .410** | .108* | .161* | .215** | **.479**** | .247** |
| SMI2 | -.072 |  | .261** | .032 | .227** | .156** | **.649**** | .208** |
| SMI3 | -.019 |  | .157** | .133** | .066 | .140** | **.709**** | .039 |
| SOI1 |  | -.088 | .037 | .570** | .065 | .165* | .221** | **.454**** |
| SOI2 |  | .086 | .045 | .198** | -.062 | .318** | .024 | **.532**** |
| SOI3 |  | .188 | -.057 | .283** | -.020 | .419** | .347** | **.483**** |

*Note*. **p* < .05; ***p* < .01; λ = standardized factor loading; ESEM = exploratory structural equation modeling; CS = compassionate self-responding; RUS = reduced uncompassionate self-responding; SSK = state self-kindness. SSJ = state self-judgment. SCH = state common humanity. SIS = state isolation. SMI = state mindfulness. SOI = state overidentification. Target loadings are bolded. Items of the Self-Judgment, Isolation, and Over-Identification subscales were reverse-coded.

**Table S4**

*Pearson's Correlations Between Study Variables in Study 1*

|  | 1 | 2 | 3 | 4 | 5 | 6 | 7 | 8 | 9 | 10 | 11 | 12 | 13 | 14 | 15 | 16 | 17 | 18 | 19 | 20 | 21 |
| --- | --- | --- | --- | --- | --- | --- | --- | --- | --- | --- | --- | --- | --- | --- | --- | --- | --- | --- | --- | --- | --- |
| 1. State SC | -- |  |  |  |  |  |  |  |  |  |  |  |  |  |  |  |  |  |  |  |  |
| 2. State SK | .650** | -- |  |  |  |  |  |  |  |  |  |  |  |  |  |  |  |  |  |  |  |
| 3. State CH | .474** | .266** | -- |  |  |  |  |  |  |  |  |  |  |  |  |  |  |  |  |  |  |
| 4. State MI | .726** | .534** | .321** | -- |  |  |  |  |  |  |  |  |  |  |  |  |  |  |  |  |  |
| 5. State SJ | .784** | .451** | .102* | .393** | -- |  |  |  |  |  |  |  |  |  |  |  |  |  |  |  |  |
| 6. State IS | .750** | .241** | .186** | .386** | .605** | -- |  |  |  |  |  |  |  |  |  |  |  |  |  |  |  |
| 7. State OI | .708** | .221** | .064 | .399** | .639** | .573** | -- |  |  |  |  |  |  |  |  |  |  |  |  |  |  |
| 8. Controllability appraisal | .586** | .414** | .241** | .558** | .423** | .395** | .384** | -- |  |  |  |  |  |  |  |  |  |  |  |  |  |
| 9. Threat appraisal | -.260** | -.003 | -.069 | -.125** | -.261** | -.316** | -.264** | -.201** | -- |  |  |  |  |  |  |  |  |  |  |  |  |
| 10. Importance appraisal | .021 | .099* | .095* | .123** | -.073 | -.060 | -.080 | .117** | .255** | -- |  |  |  |  |  |  |  |  |  |  |  |
| 11. Positive affect | .256** | .311** | .189** | .357** | .090* | .091* | .038 | .353** | -.110** | .146** | -- |  |  |  |  |  |  |  |  |  |  |
| 12. Negative affect | -.476** | -.141** | -.018 | -.250** | -.503** | -.475** | -.541** | -.340** | .308** | .100* | .103** | -- |  |  |  |  |  |  |  |  |  |
| 13. Demerits of SC | -.440** | -.259** | -.011 | -.187** | -.511** | -.419** | -.394** | -.274** | .085* | .002 | -.014 | .402** | -- |  |  |  |  |  |  |  |  |
| 14. Miserable with SC | -.581** | -.373** | -.127** | -.323** | -.593** | -.502** | -.446** | -.423** | .118** | -.071 | -.074 | .443** | .722** | -- |  |  |  |  |  |  |  |
| 15. Negative beliefs about SC | -.360** | -.178** | .078 | -.158** | -.454** | -.366** | -.379** | -.244** | .072 | -.007 | -.002 | .379** | .775** | .559** | -- |  |  |  |  |  |  |
| 16. Trait SC | .714** | .489** | .278** | .547** | .600** | .513** | .501** | .529** | -.186** | .022 | .280** | -.404** | -.470** | -.591** | -.435** | -- |  |  |  |  |  |
| 17. Trait SK | .476** | .600** | .270** | .443** | .333** | .169** | .173** | .408** | .056 | .129** | .277** | -.092* | -.269** | -.415** | -.189** | .689** | -- |  |  |  |  |
| 18. Trait CH | .316** | .302** | .477** | .308** | .104* | .072 | .053 | .238** | -.014 | .118** | .223** | .029 | .036 | -.108** | .092* | .485** | .482** | -- |  |  |  |
| 19. Trait MI | .482** | .429** | .231** | .514** | .334** | .237** | .262** | .409** | -.036 | .096* | .282** | -.143** | -.156** | -.346** | -.120** | .703** | .647** | .463** | -- |  |  |
| 20. Trait SJ | .497** | .276** | .028 | .248** | .578** | .461** | .423** | .301** | -.159** | -.056 | .060 | -.445** | -.556** | -.540** | -.553** | .702** | .308** | -.003 | .226** | -- |  |
| 21. Trait IS | .573** | .204** | .113** | .354** | .541** | .606** | .496** | .363** | -.252** | -.045 | .126** | -.430** | -.473** | -.529** | -.459** | .742** | .201** | .110** | .329** | .604** | -- |
| 22. Trait OI | .471** | .127** | .030 | .327** | .442** | .441** | .550** | .383** | -.323** | -.139** | .169** | -.475** | -.367** | -.350** | -.415** | .633** | .102* | -.013 | .216** | .539** | .627** |

*Note*. **p* < .05, ***p* < .01; SC = self-compassion. SK = self-kindness. SJ = self-judgment. CH = common humanity. IS = isolation. MI = mindfulness. OI = overidentification. Items of the state/trait self-judgment, isolation, and over-identification subscales were reverse-coded.

**Table S5**

*Standardized Factor Loadings for the Bifactor ESEM Solution for the Japanese Version of the State Self-Compassion Scale at Pretest in Study 2*

|  | SC (λ) | SK (λ) | SJ (λ) | CH (λ) | IS (λ) | MI (λ) | OI (λ) |
| --- | --- | --- | --- | --- | --- | --- | --- |
| SSK1 | **.601**** | **.522**** | .046 | .080** | -.024 | .023 | -.083** |
| SSK2 | **.639**** | **.603**** | -.050** | .054* | -.110** | .035 | -.015 |
| SSK3 | **.592**** | **.465**** | .134** | .062* | .015 | .199** | -.158** |
| SSJ1 | **.705**** | .062* | **.553**** | -.068* | .120** | -.003 | .120** |
| SSJ2 | **.721**** | .156** | **.192*** | -.049 | -.056 | -.235** | -.072** |
| SSJ3 | **.734**** | -.085* | **.094** | -.237** | -.112** | -.078 | .028 |
| SCH1 | **.325**** | -.022 | -.057 | **.677**** | .066* | .018 | -.029 |
| SCH2 | **.406**** | .062* | -.011 | **.814**** | .005 | .097** | -.071** |
| SCH3 | **.293**** | .084** | -.027 | **.801**** | .063* | .113** | -.075** |
| SIS1 | **.531**** | .021 | .098** | .069* | **.692**** | .000 | .086** |
| SIS2 | **.569**** | -.063 | -.191** | .030 | **.288**** | -.063 | .164** |
| SIS3 | **.686**** | -.119** | .056 | .075** | **.573**** | -.024 | -.045 |
| SMI1 | **.709**** | .030 | -.078* | .030 | -.031 | **.336**** | -.021 |
| SMI2 | **.598**** | .095** | -.059* | .183** | -.006 | **.601**** | .042 |
| SMI3 | **.503**** | .122** | .017 | .129** | -.036 | **.480**** | .085** |
| SOI1 | **.648**** | -.034 | .146** | -.041 | .107** | .160** | **.472**** |
| SOI2 | **.646**** | -.152** | -.068 | -.103** | .057 | -.110 | **.320**** |
| SOI3 | **.479**** | -.102 | .013 | -.144** | .020 | .030 | **.599**** |

*Note*. **p* < .05; ***p* < .01; λ = standardized factor loading; ESEM = exploratory structural equation modeling; SC= self-compassion; SSK = state self-kindness. SSJ = state self-judgment. SCH = state common humanity. SIS = state isolation. SMI = state mindfulness. SOI = state overidentification. Target loadings are bolded. Items of the Self-Judgment, Isolation, and Over-Identification subscales were reverse-coded.

**Table S6**

*Standardized Factor Loadings for the Bifactor ESEM Solution for the Japanese Version of the State Self-Compassion Scale at Posttest in Study 2*

|  | SC (λ) | SK (λ) | SJ (λ) | CH (λ) | IS (λ) | MI (λ) | OI (λ) |
| --- | --- | --- | --- | --- | --- | --- | --- |
| SSK1 | **.709**** | **.559**** | .018 | .125** | -.005 | .011 | -.009 |
| SSK2 | **.757**** | **.549**** | .029 | .011 | -.061** | .065** | -.071** |
| SSK3 | **.747**** | **.362**** | .017 | .063* | .003 | .095** | -.139** |
| SSJ1 | **.763**** | .067* | **.547**** | -.105** | .048 | -.052 | .118** |
| SSJ2 | **.885**** | -.007 | **.073** | -.114** | -.037 | -.241** | -.149** |
| SSJ3 | **.775**** | -.048* | **.095**** | -.077** | -.035 | -.021 | .136** |
| SCH1 | **.494**** | .115** | -.157** | **.620**** | .053* | .011 | .034 |
| SCH2 | **.535**** | .007 | .048 | **.793**** | -.013 | .059** | -.091** |
| SCH3 | **.535**** | .031 | -.019 | **.694**** | .027 | .122** | -.086** |
| SIS1 | **.758**** | -.017 | .072** | .042* | **.540**** | -.040* | .014 |
| SIS2 | **.698**** | -.050 | -.080* | -.007 | **.225**** | .008 | .166** |
| SIS3 | **.766**** | -.024 | -.001 | .033 | **.610**** | .015 | -.015 |
| SMI1 | **.760**** | .100** | -.042 | .084** | -.014 | **.288**** | .019 |
| SMI2 | **.705**** | .035 | -.071** | .151** | -.036 | **.516**** | .050* |
| SMI3 | **.617**** | .063* | .002 | .078** | .026 | **.463**** | .020 |
| SOI1 | **.699**** | .001 | .190** | -.055* | .090** | .091** | **.414**** |
| SOI2 | **.686**** | -.157** | -.060 | -.111** | -.014 | -.044 | **.306**** |
| SOI3 | **.617**** | -.084** | .011 | -.097** | .005 | .030 | **.554**** |

*Note*. **p* < .05; ***p* < .01; λ = standardized factor loading; ESEM = exploratory structural equation modeling; SC= self-compassion; SK = self-kindness; SJ = self-judgment; CH = common humanity; IS = isolation; MI = mindfulness; OI = overidentification. Target loadings are bolded. Items of the Self-Judgment, Isolation, and Over-Identification subscales were reverse-coded.

**Figure S1**

*Graphical Depiction of the Alternative Self-Compassion Representations*


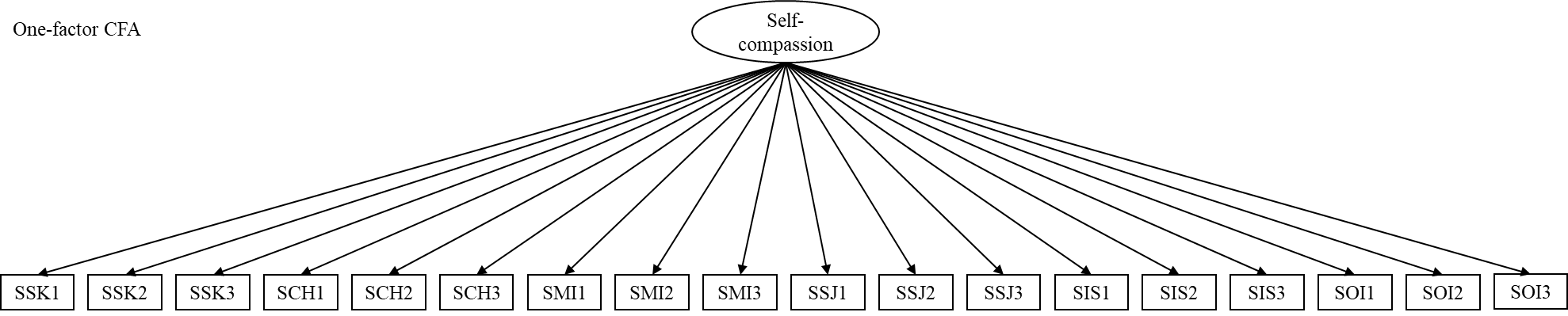


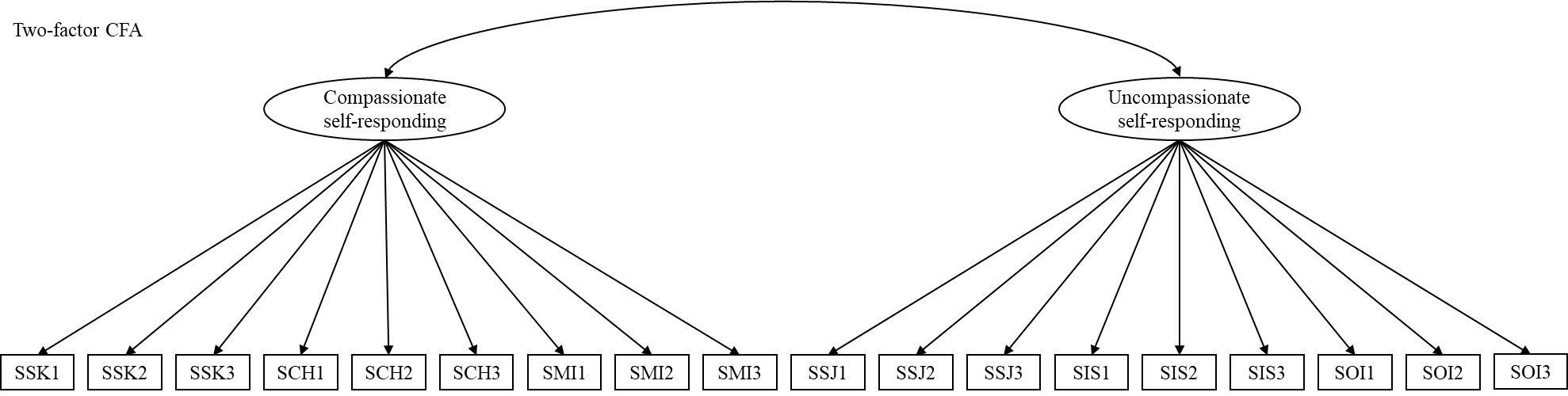


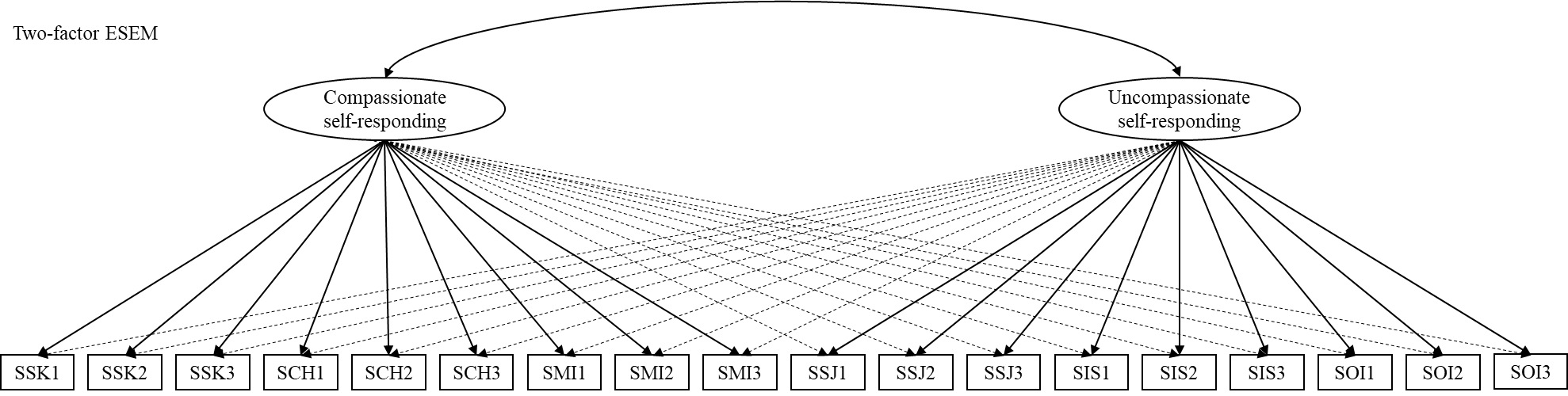


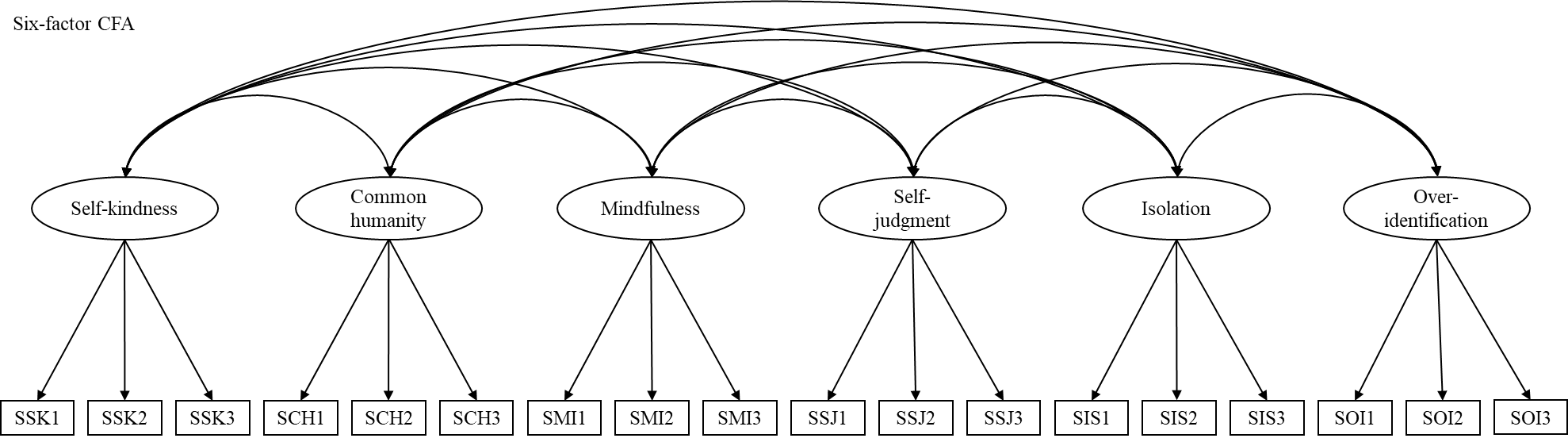


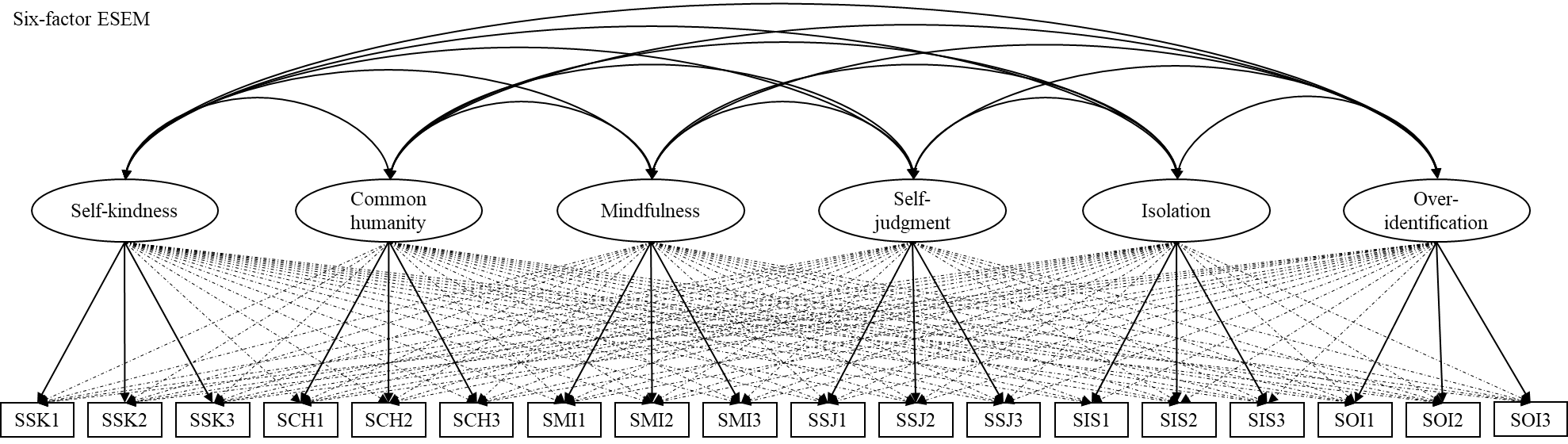


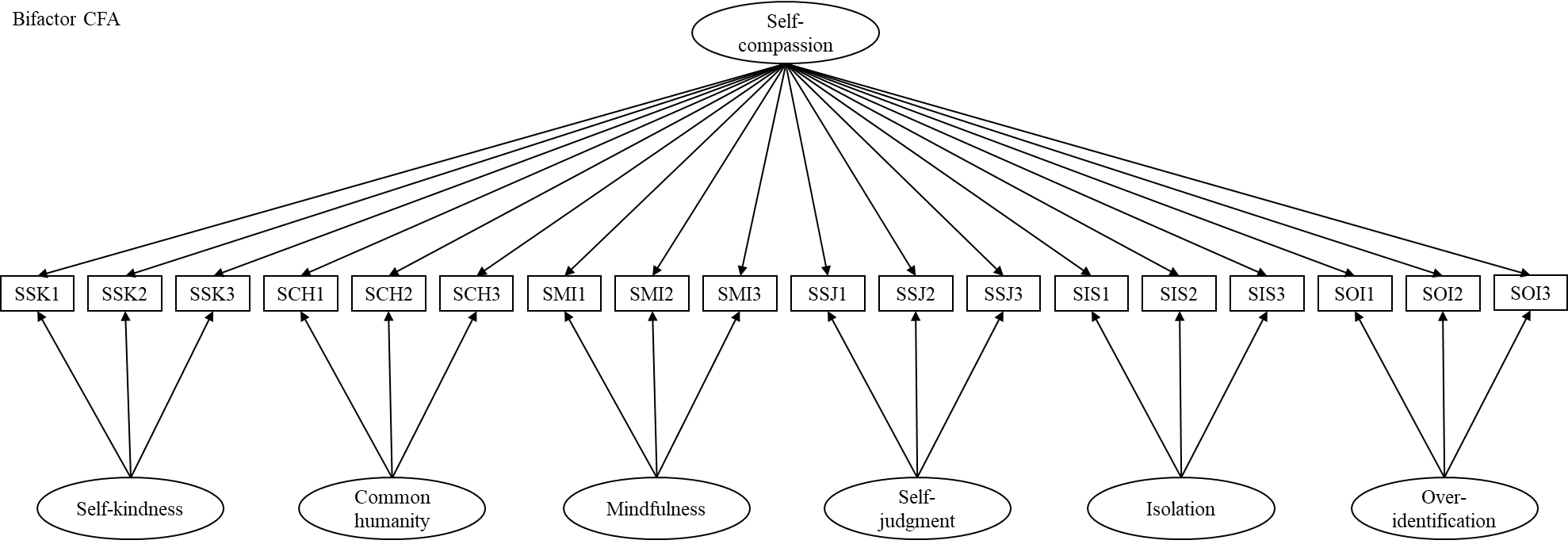


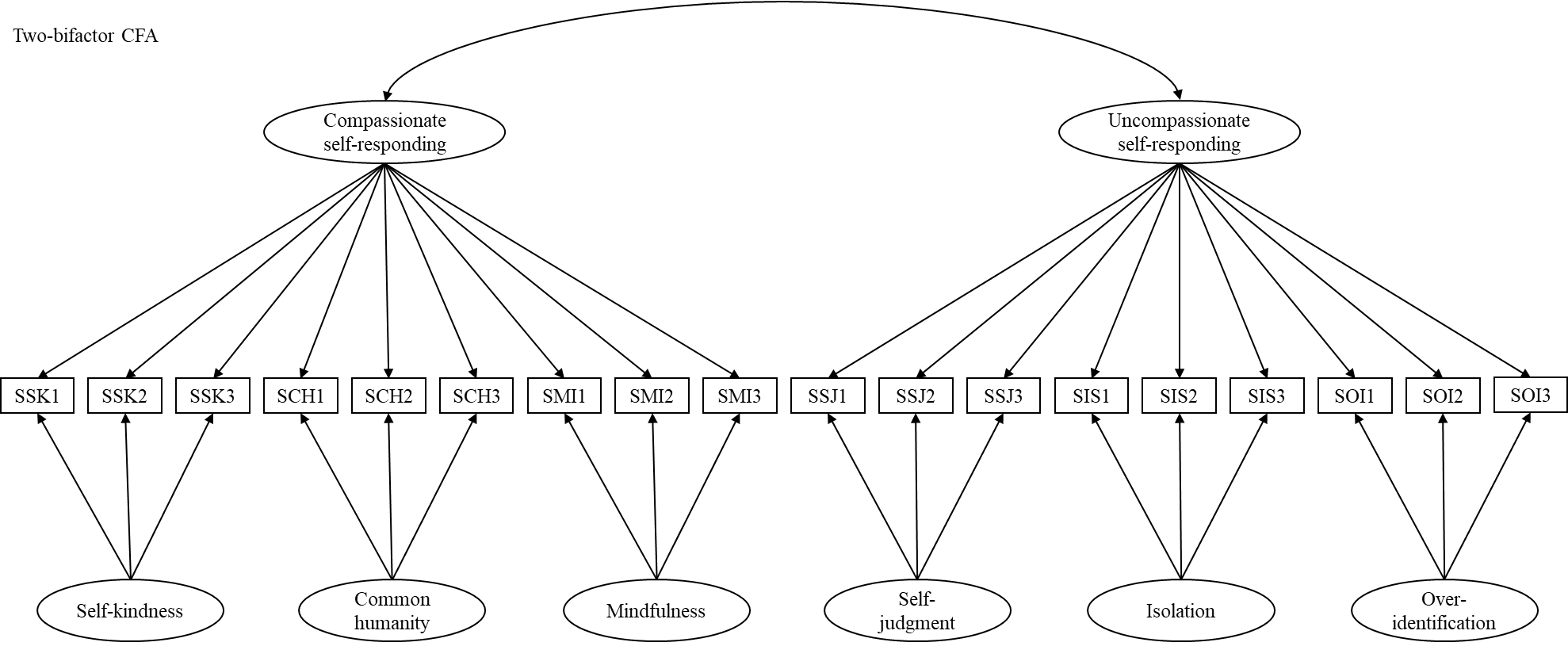


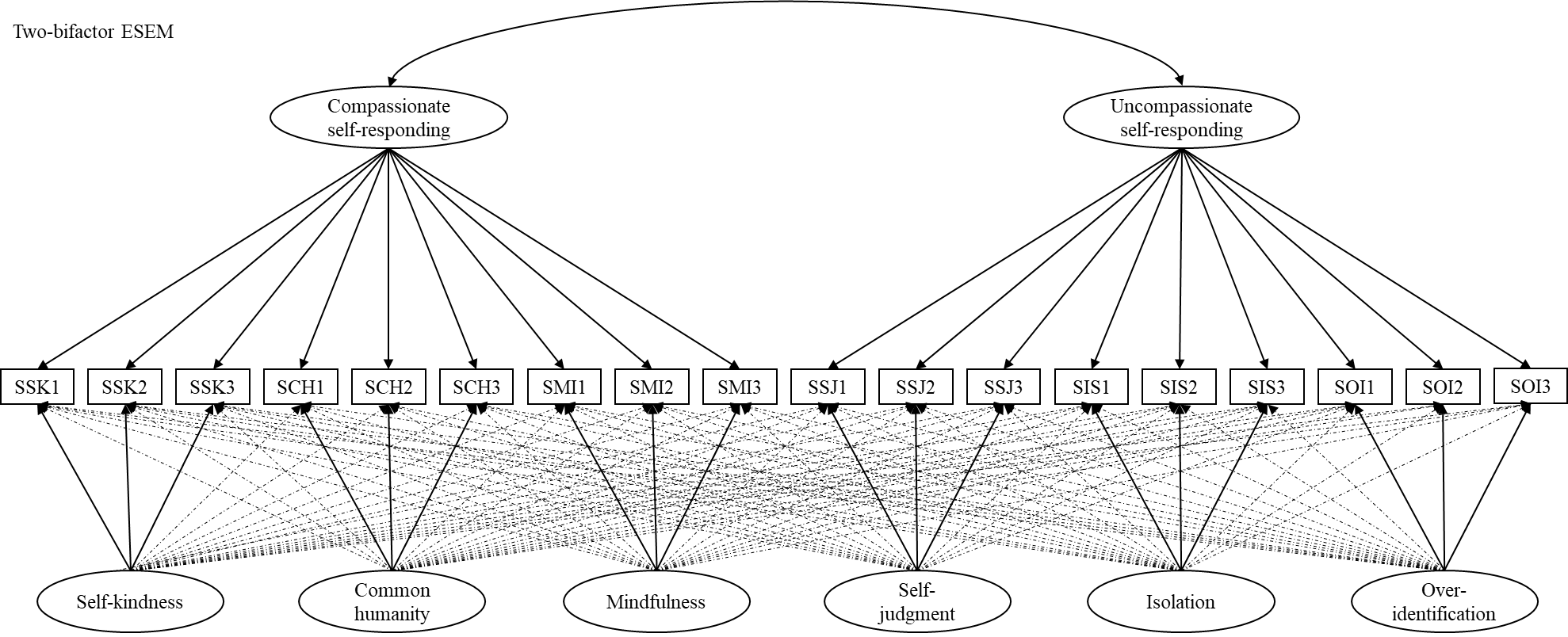


*Note*. SSK = state self-kindness. SSJ = state self-judgment. SCH = state common humanity. SIS = state isolation. SMI = state mindfulness. SOI = state over-identification. Ovals represent latent variables and squares represent questionnaire items. Unidirectional solid lines represent target paths, unidimensional dashed lines represent cross-loadings, bidirectional lines represent factor correlations.

**Appendix 1**

*The Japanese Version of the Self-Compassionate Mindstate Induction (SCMI) and the Control Writing*

**SCMI condition**

これからあなたに簡単なエクササイズに取り組んで頂きます。あなたの思い起こしている痛ましくつらい出来事に対処する際に、このエクササイズが役立つかどうかみてみましょう。

今から示す説明に可能な限り沿って、簡単な筆記課題に取り組んでください。あなたが選んだつらい状況に関して、今この瞬間にあなたの心の中で浮かび上がってくる思いや気持ちを以下のスペースに記述してください。

ストレス、恥、悲しみ、不安などのあなたが感じている不快な気持ちにも意識を向けてみましょう。そのような気持ちに意識を向けて書き出す際には、「ダメだ」や「悪い」といった評価を下さず、あなたが感じていることを「ありのままに」受け止めながら、確認していきましょう。あなたの気持ちを実際よりも軽視したり、逆に強調しすぎたりもしないようにしましょう。

例えば、「夜遅くまで恋人と通話していることを家族が迷惑だと言っていることに動揺している。恋人と話したいと思うことは普通のことだと思う。恋人との予定が夜しか合わないから仕方ないのだけれども、家族のことを思うと申し訳なさも感じる。今この瞬間もこの出来事はとてもつらいものだと思う。」

あなたの記述は匿名です。個人が特定される形で公開されることはありません。誤字脱字、文章の構造、文法は気にしないでください。

*筆記するスペースをここに*

このような状況に遭遇している他者を想像してください。このような状況にある他者とあなたはどのように同じ気持ちを共有しているかを考えてみましょう。そのような考え方を以下のスペースに記述してください。

つらい状況を経験することは人間らしさの証であり、あなたは決して独りではないと考えてみてください。苦労の仕方やそのような出来事の多さは人それぞれ異なりますが、誰もが人生でつらい出来事に直面します。あなたの経験していることは異常なことではなく、人としての人生の一部分といえます。

例えば、「このような家族関係や恋愛関係で悩んでいるのは私だけではない。人というものは，このような困難な状況の乗り越え方を学んでいくものである。多くの人は、家族関係と恋愛関係のバランスの取り方で悩むことがある。私だけではないんだ。」

あなたの記述は匿名です。個人が特定される形で公開されることはありません。誤字脱字、文章の構造、文法は気にしないでください。

*筆記するスペースをここに*

今この瞬間に聴くと役立ちそうな、ご自身の励みになり、あなたの心に温かく寄り添う言葉はどのような言葉でしょうか。以下のスペースに記述してください。

ご自身に何を言うべきか悩む場合は、同じようなつらい状況にもがき苦しんでいる身近な友人に対して、あなたがかける言葉を想像してみてください。あなたの思いやりやサポート、あなたが評価を下さないで理解していることを相手に伝えるために、あなたはどのような言葉をかけますか。その言葉が自分に語り掛ける言葉のヒントとなるか見てみましょう。

例えば、「あなたは今できることをやっていると思うよ。家族と恋人のことでうまくいかないと感じていることを本当に心苦しく思うよ。うまく折り合いをつけることができることを願うよ。あなたがこの状況を乗り越えられるように、あなたのことを支えていくよ。」

あなたの記述は匿名です。個人が特定される形で公開されることはありません。誤字脱字、文章の構造、文法は気にしないでください。

*筆記するスペースをここに*

しばらく時間を使って、ご自身に対して書いた内容を読んでみてください。ご自身に向けたやさしさや心遣いの言葉を聞いてどのように感じていますか。あなたが書いた言葉の中で、あなたの心を落ち着かせて、あなたの役に立つような言葉があるか注目してみてください。ご自身の言葉を読む際に、ゆっくりと深呼吸を2、3回してください。ご自身からのサポートを受け取ってみましょう。

**Control condition**

これからあなたに簡単なエクササイズに取り組んで頂きます。あなたの思い起こしている痛ましくつらい出来事に対処する際に、このエクササイズが役立つかどうかみてみましょう。

今から示す説明に可能な限り沿って、簡単な筆記課題に取り組んでください。そのつらい状況で実際に何が起きているのかについて、できるだけ具体的に、以下のスペースに記述してください。

例えば、「私が夜遅くまで恋人と通話していることで家族ともめている。私は恋人と通話していたいのに、母はそのことを良く思っていない。なぜなら母は～。」

あなたの記述は匿名です。個人が特定される形で公開されることはありません。誤字脱字、文章の構造、文法は気にしないでください。

*筆記するスペースをここに*

その状況にあなた以外の他者が関わっているのならば、誰が関わっているのかを記述してください。関わっている人々のことをできる限り詳細に説明してください。もしあなただけがその状況に関わっている場合はご自身について説明してください。

例えば、「家族とのもめごとには、母、姉、弟が関わっていて、母と姉は私のことを理解してくれない。弟は私が恋人と夜に通話することに理解を示してくれている。姉は2歳年上で、弟は1歳年下です。」

あなたの記述は匿名です。個人が特定される形で公開されることはありません。誤字脱字、文章の構造、文法は気にしないでください。

*筆記するスペースをここに*

あなたがご自身に向けて心の中で言ったこと、他者があなたに言ったこと、あなたが他者に言ったことなど、その状況で話された言葉について、できる限り詳細に記述してください。

例えば、「私は母に「恋人と予定があうのが夜の時間帯だけだから、話せるときにゆっくりと話したい」と言いました。けれども母は「話し声が寝室まで聞こえて、眠れない。家族のことをもっと考えるべきだ」と私に言いました。」

あなたの記述は匿名です。個人が特定される形で公開されることはありません。誤字脱字、文章の構造、文法は気にしないでください。

*筆記するスペースをここに*

しばらく時間を使って、あなたが書いた内容を読んでみてください。ご自身にとって特に印象的なものはありますか。
